# Genetic dissection of stem and leaf rachis prickles in diploid rose using a pedigree-based QTL analysis

**DOI:** 10.3389/fpls.2024.1356750

**Published:** 2024-09-18

**Authors:** Zena J. Rawandoozi, Andrew Barocco, Maad Y. Rawandoozi, Patricia E. Klein, David H. Byrne, Oscar Riera-Lizarazu

**Affiliations:** ^1^ Department of Horticultural Sciences, Texas A&M University, College Station, TX, United States; ^2^ Norman Borlaug Institute for International Agriculture and Development, Texas A&M AgriLife Research, Texas A&M System, College Station, TX, United States

**Keywords:** FlexQTL, haplotype, prickle density, QTL, *Rosa*

## Abstract

**Introduction:**

Prickles are often deemed undesirable traits in many crops, including roses (*Rosa* sp.), and there is demand for rose cultivars with no or very few prickles. This study aims to identify new and/or validate reported quantitative trait loci (QTLs) associated with stem and leaf rachis prickle density, characterize the effects of functional haplotypes for major QTLs, and identify the sources of QTL-alleles associated with increased/decreased prickle density in roses.

**Methods:**

QTL mapping using pedigree-based analysis (PBA), and haplotype analysis were conducted on two multi-parental diploid rose populations (TX2WOB and TX2WSE).

**Results and discussion:**

Twelve QTLs were identified on linkage groups (LGs) 2, 3, 4, and 6. The major QTLs for the stem prickle density were located between 42.25 and 45.66 Mbp on chromosome 3 of the *Rosa chinensis* genome assembly, with individual QTLs explaining 18 to 49% of phenotypic variance (PVE). The remaining mapped QTLs were minor. As for the rachis prickle density, several QTLs were detected on LG3, 4, and 6 with PVE 8 to 17%. Also, this study identified that ancestors *R. wichurana* ‘Basye’s Thornless’, ‘Old Blush’, and the pollen parent of M4-4 were common sources of favorable alleles (*q*) associated with decreased prickle density, whereas ’Little Chief’ and ‘Srche Europy’ were the source of unfavorable alleles (*Q*) in the TX2WOB and TX2WSE populations, respectively. The outcomes of this work complement other studies to locate factors that affect prickle density. These results can also be utilized to develop high-throughput DNA tests and apply parental selection to develop prickle-free rose cultivars.

## Introduction

Prickles are a common form of plant defense used in response to a broad range of biotic and abiotic stresses (Kellogg et al., 2011). Prickles are widely present and exhibit great diversity in shape, color, size, and density across different plant species, especially among the rose family (Rosaceae) (Khadgi and Weber, 2020; Zhou et al., 2021). Rose (*Rosa* sp.) is a commercially important flower crop worldwide, and approximately 35,000 commercial cultivars have been described (Blechert and Debener, 2005). Roses can be found as bushes, shrubs, and climbing plants, and most have persistent prickles (Burns, 2014), creating challenges for the rose industry. Prickle removal is a crucial process before packaging in cut rose production, as it can lead to stem wounds, negatively affecting transportation tolerance and ornamental value. Furthermore, prickles can cause injuries to workers during harvesting and handling. Consequently, roses with many prickles are generally not preferred for cut rose production, even with outstanding ornamental features (Zhou et al., 2021).

According to previous studies, the absence of prickles in rose mutants has been either a transient or a stress-sensitive trait. Rose breeders have observed that prickless mutants lost their stability over time (Nobbs, 1984; Rosu et al., 1995; Singh et al., 2017), and prickly phenotypes reappeared in response to environmental stressors (Nobbs, 1984; Canli and Skirvin, 2003). The precise morphogenetic and molecular mechanisms underlying prickle development are not well understood. Prickles have been hypothesized to originate from multiple cellular divisions of the epidermis and are considered modified glandular trichomes (Kellogg et al., 2011; Pandey et al., 2018; Feng et al., 2021). A recent hypothesis proposes that prickles originate from either glandular or non-glandular structures, leading to glandular and non-glandular prickles, respectively (Zhou et al., 2020). The presence of prickles was reported to be controlled by a single dominant gene or complementary genes (prickles dominant to no prickles) (Debener, 1999; Crespel et al., 2002; Shupert et al., 2007). However, the density of stem prickles appears to be quantitatively controlled by several genes. Previous QTL mapping studies indicate one to two major QTLs for prickle density on linkage group (LG) 3 across various diploid and tetraploid populations, with minor QTL effects observed on other LGs (Crespel et al., 2002; Linde et al., 2006; Koning-Boucoiran et al., 2009; Bourke et al., 2018; Hibrand Saint-Oyant et al., 2018; Zhou et al., 2020; Zhong et al., 2021).

The high broad-sense heritability (H^2^) and low genotype by environment (G×E) interaction were reported for stem and petiole (leaf rachis) prickles (Gitonga et al., 2014). Previous studies observed a low correlation between stem and petiole prickles, suggesting that the two traits segregate independently (Rajapakse et al., 2001; Gitonga et al., 2014). Rajapakse et al. (2001) reported that separate genes controlled prickles on the stem and petioles. Also, a major QTL for prickle density on petiole was mapped on LG4 (Bourke et al., 2018).

Feng et al. (2015) suggested that the RcTTG1(TRANSPARENT TESTA GLABRA1) gene located at ~63.98 Mbp on chromosome 1 on the *Rosa chinensis* Genome v1.0 may influence and regulate the formation of rose prickles (Hibrand Saint-Oyant et al., 2018; Zhou et al., 2020). TTG1 is a WD40 transcription factor that is reported to control the development of the trichomes in *Arabidopsis* (*A. thaliana*) (Walker et al., 1999; Huang et al., 2022). Hibrand Saint-Oyant et al. (2018) proposed that the RcTTG2 located at ~33.40 Mbp on chromosome 3 on the *Rosa chinensis* Genome v1.0 might be a good candidate gene for controlling prickle development in roses. TTG2 is a WRKY transcription factor involved in trichome development in *Arabidopsis* (Johnson et al., 2002; Liu et al., 2024).

This study seeks genetic determinants of prickle density utilizing multi-parental populations and pedigree-based QTL analysis using FlexQTL (Bink et al., 2014). This method provides a wider genetic sampling and enhanced QTL detection, especially for shared QTL, and evaluates allelic effects across various genetic backgrounds. FlexQTL also enables marker haplotype phasing and traces allele origins via identity-by-descent probabilities, and it has been used in multiple Rosaceous crops (Bink et al., 2014; Verma et al., 2017; Rymenants et al., 2020; Kostick et al., 2021; Rawandoozi et al., 2021; Crump et al., 2022; da Silva Linge et al., 2024).

This study aims to (1) identify and validate QTLs linked to stem and rachis prickles in two multi-parental diploid populations; (2) examine functional haplotype effects on major QTLs; and (3) identify the sources of QTL-alleles associated with the increase/decrease in prickle density in roses. Understanding the genetic basis for prickles will aid the design of strategies to develop rose cultivars with varying levels of pickle density or no prickles. Prick-free varieties should allow better handling during cultivation and processing. In addition, the results of this study can contribute to the ongoing efforts to enhance the efficiency of rose breeding programs worldwide.

## Materials and methods

### Plant materials

Two multi-parental diploid rose populations [TX2WOB (298 progenies) and TX2WSE (355 progenies)] were phenotyped for prickle density in the stem and leaf rachis in 2021 in research fields in Texas (**Supplementary Table 1**). These two diploid rose populations were created by the Texas A&M Rose Breeding and Genetics Program and used in previous studies (Dong et al., 2017; Liang et al., 2017b; 2017a; Kang et al., 2019; Wu et al., 2019; Young, 2020; Rawandoozi et al., 2022; Young et al., 2022; Rawandoozi et al., 2023).

**Figure 1:**
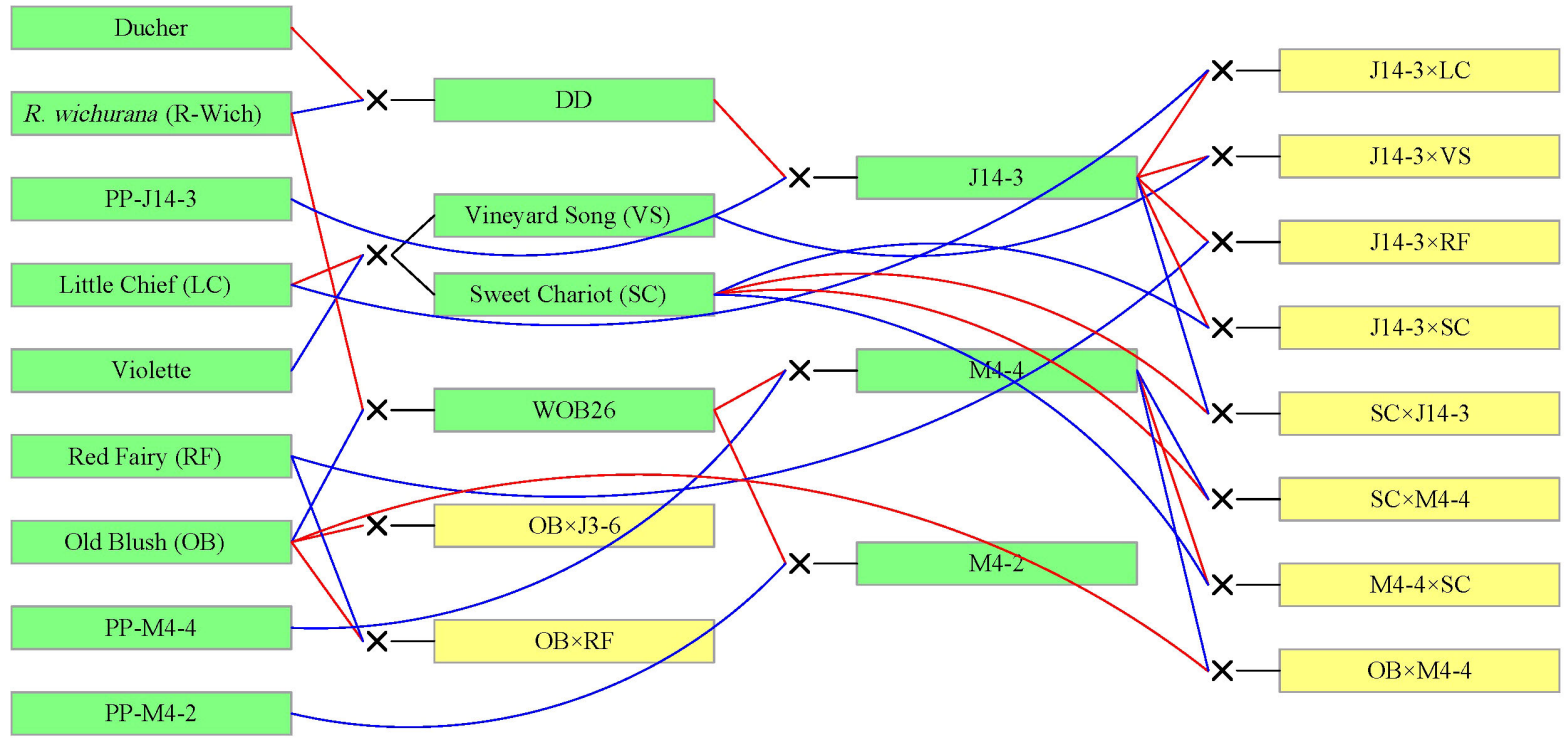
Pedigree of the TX2WOB multi-parental population composed of 10 F_1_ diploid rose families derived from intercrossing eight parents. Red and blue lines link progeny to female and male parents, respectively, generated using Pedimap 1.2.

**Figure 2:**
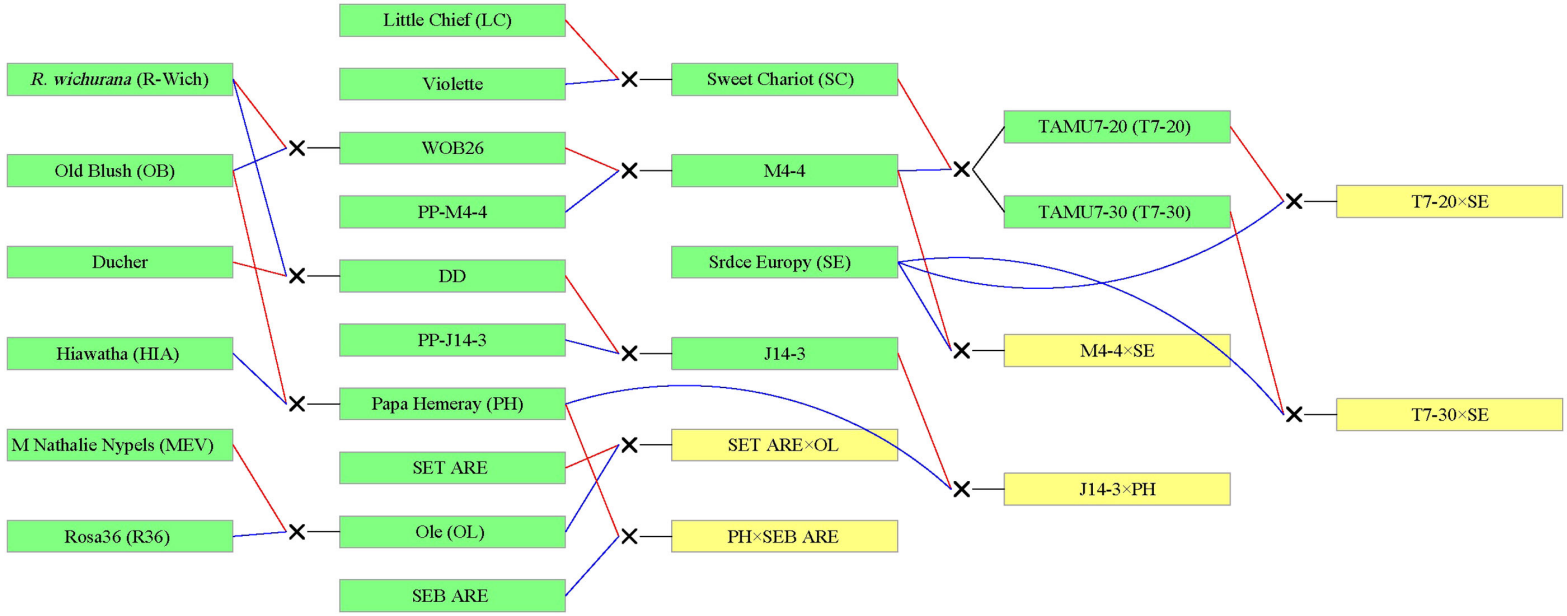
Pedigree of the TX2WSE multi-parental population composed of six F_1_ diploid rose families derived from nine parents. Red and blue lines link progeny to female and male parents, respectively, generated using Pedimap 1.2. SET-ARE, *R. setigera*-ARE; SEB-ARE, Swamp Rose EB-ARE.

TX2WOB consists of 10 F_1_ families (**Figure 1**), and TX2WSE comprises six F_1_ families (**Figure 2**). Both populations were derived from *R. wichurana* ‘Basye’s Thornless’ (‘R-Wich’) and ‘Old Blush’ (‘OB’), ‘Little Chief’ (‘LC’), ‘Red Fairy’ (‘RF’), ‘Sweet Chariot’ (‘SC’), and ‘Vineyard Song’ (‘VS’). The TX2WSE populations are also encompassed ‘Papa Hemeray’ (‘PH’) and ‘Srdce Europy’ (‘SE’) parents. Glabrous and prickly roses were present in both populations with diverse prickle densities, sizes, shapes, and colors (**Supplementary Figures 1–3**). Glabrous roses like ‘R-Wich’ and the Texas A&M breeding line M4-4, are present in both populations, and *R. setigera*-ARE (SET-ARE) is present only in TX2WSE, whereas the rest are known to be prickly roses. In general, TX2WSE has more prickly stem parents in their background than the TX2WOB.

In 2018, plants of the TX2WOB and TX2WSE populations were planted in a randomized complete block design with two replications (where individual plants were the experimental unit) at the Texas A&M University Horticulture Teaching Research and Extension Center (HortTREC) in Somerville, TX (30.524591, −96.422479). More details on populations and field conditions are described by Rawandoozi et al. (2022).

### Phenotyping for stem and rachis prickles

This research identified a prickle as any sharp protrusion from the stem or rachis that exceeds ½ mm in length. The stem prickles density was assessed on three mature canes by counting prickles over a 15-cm midsection, equidistant from the crown and apex. For rachis prickle density, one mature leaf per cane was chosen, and prickles were counted from the leaf’s base (the point where the petiole attaches to the stem) to the rachis end.

### Genotyping and consensus map development

Genomic DNA from young rose leaves was extracted using Doyle’s CTAB protocol (Doyle and Doyle, 1991). GBS was conducted with the *Ngo*MIV restriction enzyme, following the methods described by Morishige et al. (2013). Single-end sequencing was conducted on the templates using Illumina HiSeq 2500 following Illumina protocols and initially filtered with FastQC (Illumina). Reads were then sorted by barcode using a custom Python script; only reads with an exact match to both the barcode and the partial NgoMIV restriction site proceeded through the pipeline. Barcodes were trimmed, and the 113-bp reads were aligned to the *Rosa chinensis* v1.0 genome (Hibrand Saint-Oyant et al., 2018) using CLC Genomics Workbench v9.0 (Qiagen, Boston, MA). Alignment parameters included: a mismatch cost of 2, an insertion and deletion cost of 3, a minimum read length of 50% required to match the reference, and at least 75% similarity between reads and the reference genome. Reads that either did not align or aligned to multiple locations were excluded. SNP detection was conducted using the Variant Detection Tool in CLC Genomics Workbench, adhering to specific parameters: a detection probability of 90%, a minimum read coverage of 15, at least three SNP counts, a neighborhood radius of 5, minimum central quality of 20, and minimum neighborhood quality of 15. The resulting mapping and SNP files were exported in SAM and comma-separated values (CSV) formats, respectively. Furthermore, SNP call analysis was conducted using custom Python and Perl scripts. Markers were named based on their physical positions in the rose genome, and alleles were converted to the CP population segregation types described in the JoinMap^®^ v5.0 manual (www.kyazma.nl) using a custom Python script. Markers were grouped into bins based on their proximity to a restriction enzyme cut site in the reference genome, a process referred to as REbinning. Between 400,000 and 180,000 SNPs were identified for the two datasets of diploid rose populations: TX2WOB (five families, 415 individuals) and TX2WSE (three families, 314 individuals).

Prior to developing two consensus maps, for TX2WOB, low-quality SNP markers were removed before developing the five individual linkage maps (Rawandoozi et al., 2022). Using TASSEL v5, markers were excluded if they were mapped to chromosome 0, were non-biallelic, or had more than 10% missing data. A Microsoft Excel-based tool and custom R scripts were then used to eliminate markers with inheritance errors. After curation, nearly 90,502 SNP markers were employed for constructing the integrated consensus map (ICM) for TX2WOB (**Supplementary Table 2**). The R package “polymapR” v. 1.1.1 was utilized to create individual maps for each population, removing duplicated and distorted markers (p ≥ 0.001). The datasets were streamlined by selecting one marker per restriction-enzyme bin around a NgoMIV cut site, prioritizing markers common between populations, with minimal missing data, and fitting expected segregation ratios. The consensus map was then developed using the R package “LPmerge” v. 1.7, and visualized with “LinkageMapView” v. 2.1.2 and MapChart v. 2.32.

As for TX2WSE, nearly 58,000 SNP markers were utilized for constructing the ICM (**Supplementary Table 3**). Similar procedures were followed to generate a linkage map, except that markers were filtered in PLINK v.1.9 to eliminate Mendelian-inconsistent errors per population (Young et al., 2022).

Before QTL analysis, further curation in FlexQTL software v.0.1.0.42 was performed to identify and fix singletons and double recombinations using the SIP_Population_6.csv and DoubleRecomb.csv files. This curation process was iterated until no errors were observed, as visualized through FlexQTL outputs. Additional curation for inheritance errors, as determined in the mconsistency.csv file from FlexQTL outputs, was also performed. A total of 1,115 and 866 SNP informative markers were kept for TX2WOB and TX2WSE populations, respectively, and used for QTL mapping.

### QTL mapping and characterization

The genotypic and phenotypic data were combined for each multi-parent population (TX2WOB and TX2WSE) and analyzed using FlexQTL, which utilizes the Bayesian analysis method to estimate the number and position of QTLs, mode (peak), and magnitude of QTL(s) in unbalanced population sets (Bink et al., 2014). For QTL analysis purposes, the phenotypic values of prickle density in the stem and rachis were averaged for individuals over the two replicates. Pairwise comparisons of models (1/0, 2/1, 3/2, etc.) using twice the natural log of the Bayes factor (2lnBF) statistic was utilized to infer the number of mapped QTLs. The statistical evidence for QTLs was assessed by 2ln(BF); values greater than 2, 5, and 10 indicate positive, strong, and decisive evidence, respectively (Kass and Raftery, 1995). Traits were initially tested using a mixed model (permitting QTLs with additive and dominant effects). As no dominance effect was detected, QTL analysis was run with an additive effect model. Markov chain Monte Carlo (MCMC) simulation lengths spanned 100,000 iterations to store at least 1,000 samples with a thinning 100 for all runs. The effective sample size (ESS) in the parameter file was set to 101 to ensure proper convergence (Bink et al., 2014). In this research, QTLs with strong (2lnBF ≥ 5) or decisive evidence (2lnBF ≥ 10) in the same genomic region across two populations and contributing at least 15% of phenotypic variation were considered major QTLs. Next, FlexQTL was used to generate a new file (MQTRRegions.info) to further refine QTL intervals, recalculating phenotypic variance explained (PVE) for the discovered QTLs, QTL intensity and mode positions, and new files (MQTRegionsGTP.csv and mhaplotypes.csv) for haplotype analysis.

From FlexQTL additive model outputs, additive variance $\left(\sigma_{A\left(trt\right)}^{2}\right)$ for the trait was calculated by subtracting the residual variance $\left(\sigma_{e}^{2}\right)$ from the phenotypic variance $\left(\sigma_{P}^{2}\right)$ . PVE was calculated as follows:


$$PVE=\frac{\sigma_{A\left(qtl\right)}^{2}}{\sigma_{P}^{2}}\times 100\;$$

where: $\sigma_{A\left(qtl\right)}^{2}$ is the additive variance of a QTL.

The narrow-sense heritability (h^2^) was estimated with the equation:


$$h^{2}=\frac{\sigma_{A\left(trt\right)}^{2}}{\sigma_{P}^{2}}$$

Our QTL nomenclature follows the conventions of the Genome Database for Rosaceae (Jung et al., 2014). Thus, the name *q*SPCK.TX2WOB-LG3.1 stands to a QTL for the stem prickles (SPCK) trait mapped in the consensus map of the multi-parent population (TX2WOB), on with the linkage group number (LG3) and a number (1 or 2) to differentiate QTLs in case there is more than one QTL on the same LG.

Haplotype analysis was conducted on SNPs within regions of major QTLs that consistently mapped across both populations with strong or decisive evidence and exhibited high PVE (≥15%). Also, for those QTLs that overlapped for both traits or showed a PVE greater than 15% in one population, haplotypes were constructed using FlexQTL and the “PediHaplotyper” v1.0 R package (Voorrips et al., 2016). To determine the statistical significance of diplotype effects, a non-parametric multiple comparison Steel–Dwass test (P < 0.05) was used in JMP Pro version 13.2 (SAS Institute Inc., Cary, NC. 2016). QTL allele genotypes (*Q* or *q*) were allocated to haplotypes based on the direction of their effects (increasing or decreasing prickles density). In instances of multiallelic series, *Q*- and *q*-alleles were assigned index numbers. Finally, *q*-/*Q*-allele sources were identified through pedigree records and classified as identical by descent (IBD) if traced to a common ancestor or identical by state (IBS) if no known common ancestor was found (Rawandoozi et al., 2022, 2023).

## Results

### Phenotypic data analysis

In this study, prickle density varied between stem (SPCK) and leaf rachis (RPCK) in both examined populations. The average prickle count for SPCK was roughly 9 to 10 prickles per 15 cm of stem length within TX2WOB and TX2WSE. Prickle counts ranged from 0.0 to 24.5 in TX2WOB and from 0.0 to 30.3 in TX2WSE (**Supplementary Table 4**). None of the data displayed a normal distribution as both population data sets skewed toward fewer prickles (**Supplementary Figure 4**). Regarding RPCK, TX2WOB had fewer rachis prickles per leaf (mean = 1.7, range 0–10) than did TX2WSE (mean = 3.9, range 0–17) (**Supplementary Table 4** and **Supplementary Figure 5**). There were weak correlations (ranging from 0.15 to 0.19) between SPCK and RPCK in both populations (**Supplementary Table 5**).

### Genome-wide QTL analysis

The narrow-sense heritability (h²) estimates from FlexQTL ranged from moderately high for SPCK (0.60–0.64) to moderately low for RPCK (0.25–0.42) (**Table 1**). Using a random effects model with restricted maximum likelihood (REML) analysis, high (0.83) and moderate (0.40) broad sense heritability (H^2^) were observed for SPCK and RPCK, respectively (data not shown).

**Table 1:** QTLs affecting prickle density on stem and rachis measured on the TX2WOB (10 F_1_ diploid rose families) and TX2WSE (six F_1_ diploid rose families) populations in 2021 in Somerville, Texas.

Prickle location	Population	Records^*^	*μ*	*σ^2^_p_*	*σ^2^_e_*	*σ^2^_A_*	*h^2^*	*LG*	*2ln(BF)*		
									1/0	2/1	3/2
Stem	TX2WOB	290	9.1	31.33	12.61	18.72	0.60	2	9.7	−1.9	NA
								3	NA	30.5	1.4
								6	15.6	−2.5	NA
	TX2WSE	304	9.9	48.28	17.26	31.02	0.64	3	NA	7.4	2.7
								6	3.7	0.0	−1.3
Rachis	TX2WOB	291	1.7	2.32	1.73	0.59	0.25	4	2.9	0.5	−1.1
								6	28.4	2.1	0.6
	TX2WSE	306	3.9	7.03	4.06	2.96	0.42	3	4.6	0.4	−0.1
								6	6.8	2.0	0.4

^*^The number of data points (Records), phenotypic mean (*μ*), phenotypic variance (*σ^2^_P_*), residual variance (*σ^2^_e_*), additive variance (*σ^2^_A_*), narrow-sense heritability (*h^2^*), the linkage groups (*LG*) that QTLs were mapped on, and Bayes Factor [*2ln(BF)*]. Bayes factor quantifies the support from the data for the number of QTL(s) in the model (QTL evidence) after pairwise model comparison (1/0, 2/1, and 3/2) such as ‘one-QTL model’ vs. ‘zero-QTL model’, etc. *2ln(BF)* <0 = no evidence; 0–2 = hardly any; 2–5 = positive; 5–10 = strong; >10 = decisive. Bayes factor is not available (na) if either model does not have enough samples in the Markov chain.

**Figure 3:**
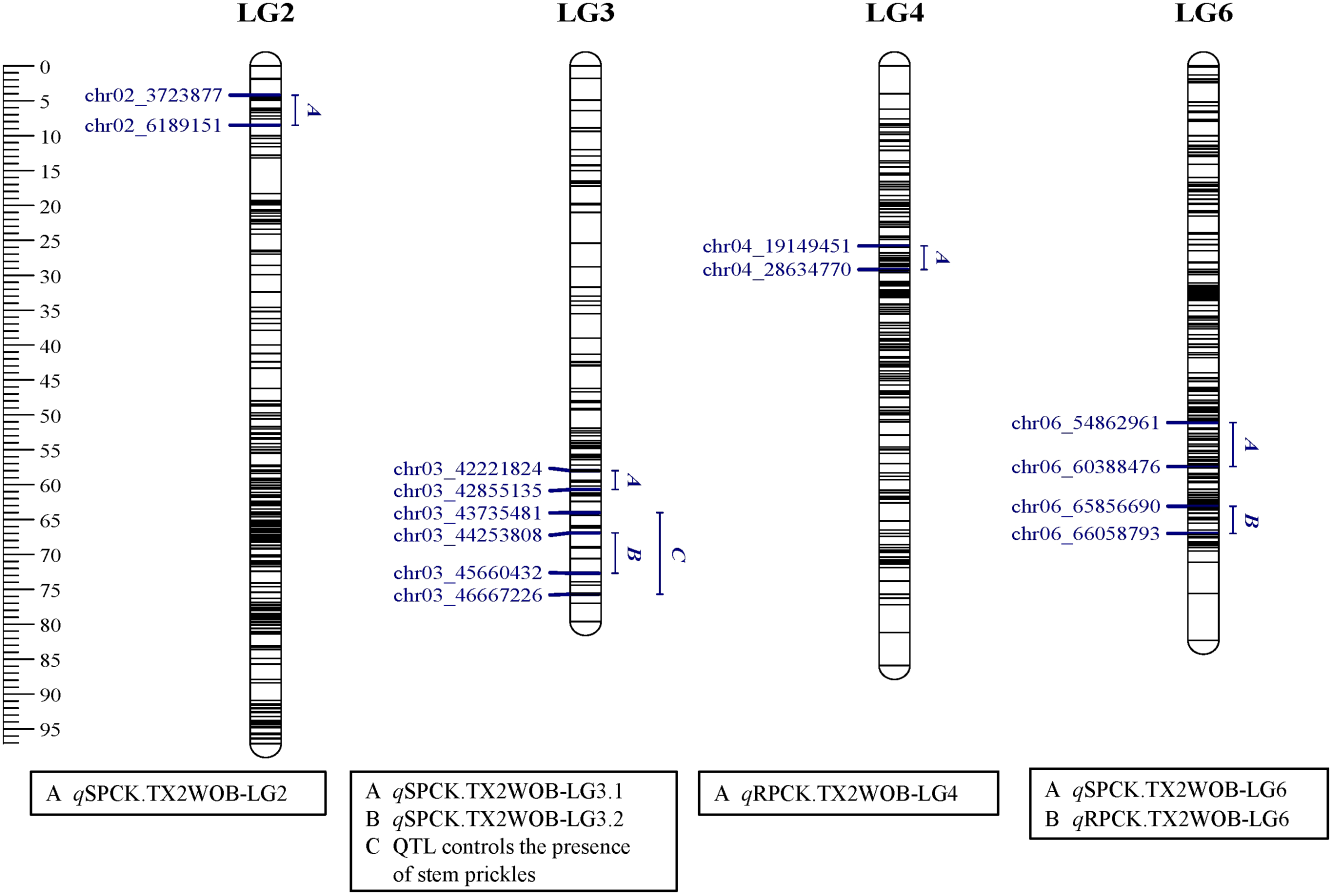
Positions of QTLs controlling prickle density on the stem (SPCK), rachis (RPCK), and QTL controlling the presence of stem prickles in the TX2WOB population. QTL names are listed below each LG. The plot was generated using MapChart 2.32.

**Figure 4:**
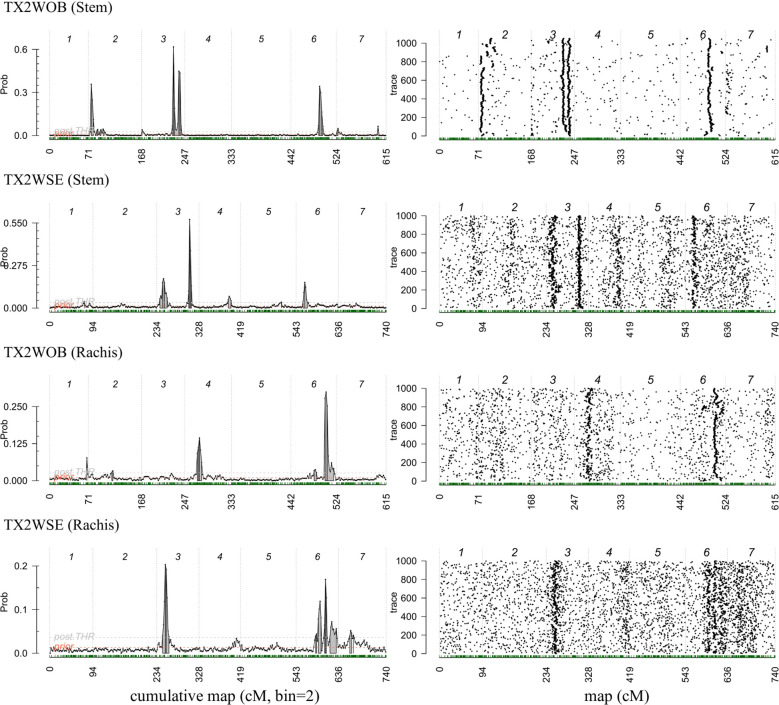
Posterior positions (left) and trace samples QTL positions (right) based on an additive model performed using Visual FlexQTL software for prickle density on the stem and rachis phenotyped in 2021 on TX2WOB and TX2WSE diploid rose populations in Somerville, Texas.

**Figure 5:**
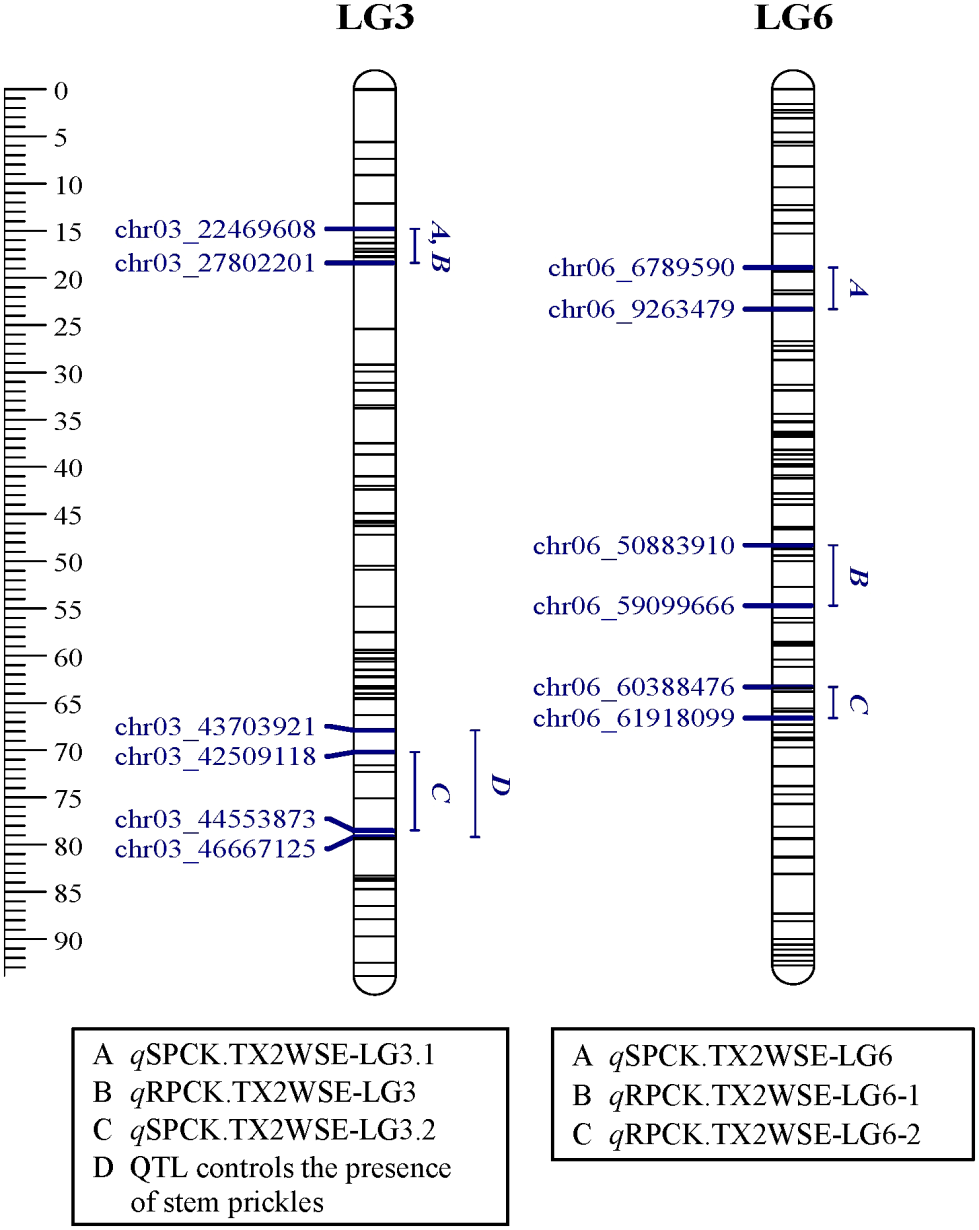
Positions of QTLs controlling prickle density on the stem (SPCK), rachis (RPCK), and QTL controlling the presence of stem prickles in the TX2WSE population. QTL names are listed below each LG. The plot was generated using MapChart 2.32.

Regarding SPCK density, four QTLs on LG2, LG3, and LG6 were identified in TX2WOB, whereas three QTLs were mapped on LG3 and LG6 in TX2WSE. As for RPCK, two QTLs on LG4 and LG6 were found in TX2WOB; meanwhile, three QTLs on LG3 and LG6 were detected in TX2WSE (**Table 1** and **Figures 3**–**5**).

In the TX2WOB analysis for SPCK, two major neighboring QTLs with decisive evidence were mapped on LG3. The first QTL, *q*SPCK.TX2WOB-LG3.1, was located between 58.0 cM and 60.7 cM (42.22 Mbp–42.85 Mbp) with peak mode at 59.6 cM and PVE was 18% (**Tables 1**, **2** and **Figure 3**). The second QTL, *q*SPCK.TX2WOB-LG3.2, was mapped with an interval between 66.9 and 72.7 cM (44.25 Mbp–45.66 Mbp) with a peak of 69.1 cM and PVE 43%. Additional QTLs on LG6 and LG2 were detected with decisive and strong evidence, respectively, and PVE up to 14%.

**Table 2:** QTL name, linkage group (LG), position of the nearest SNP marker to mode (peak), QTL interval, posterior intensity (QTL intensity), and phenotypic variance explained (PVE) for prickle density on the stem (SPCK) phenotyped on the TX2WOB (ten diploid rose families) and TX2WSE (six diploid rose families) populations in 2021 in Somerville, Texas.

QTL name	LG	Mode		Interval		QTL intensity	PVE (%)
		(cM)	(Mbp)	(cM)	(Mbp)		
*q*SPCK.TX2WOB-LG2	2	6.1	4.79	4.2	3.72	0.74	9
				8.5	6.18		
*q*SPCK.TX2WOB-LG3.1	3	59.6	42.55	58.0	42.22	0.87	18
				60.7	42.85		
*q*SPCK.TX2WOB-LG3.2	3	69.1	45.39	66.9	44.25	1.02	43
				72.7	45.66		
*q*SPCK.TX2WOB-LG6	6	53.1	56.07	51.1	54.86	0.88	14
				57.4	60.38		
*q*SPCK.TX2WSE-LG3.1	3	17.74	22.89	14.79	22.46	1.03	9
				18.39	27.80		
*q*SPCK.TX2WSE-LG3.2	3	72.26	44.46	70.20	42.50	1.33	49
				78.51	44.55		
*q*SPCK.TX2WSE-LG6	6	19.33	7.12	18.92	6.78	0.62	7
				23.26	9.26		

**Figure 6:**
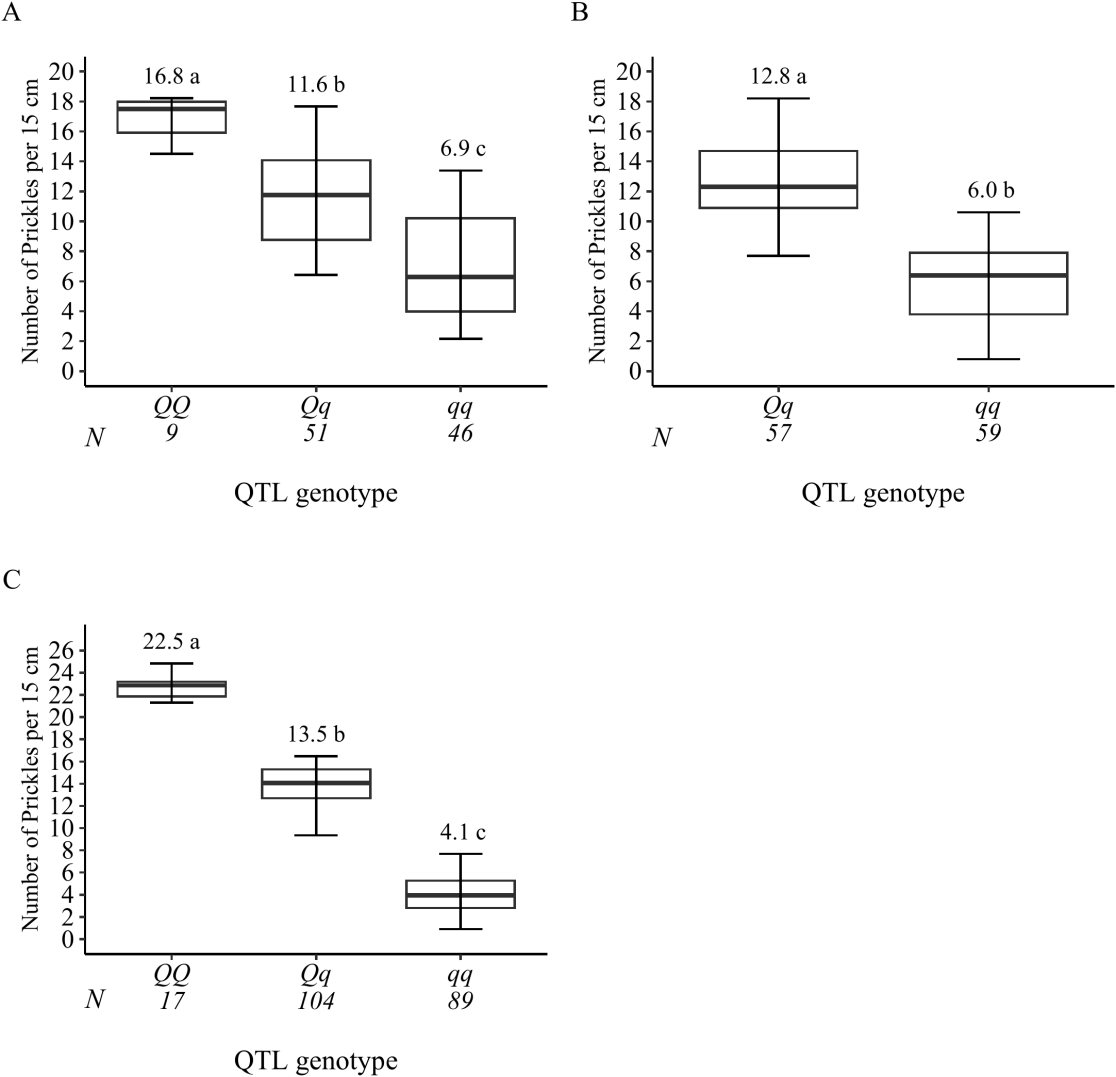
Probable QTL genotype at the signal peak from all progenies for prickle density QTLs *q*SPCK.TX2WOB-LG3.1 **(A)** and *q*SPCK.TX2WOB-LG3.2 **(B)** of the diploid rose population TX2WOB, and *q*SPCK.TX2WSE-LG3.2 **(C)** of TX2WSE. Means not connected by the same letter are significantly different (p<0.05).

**Figure 7:**
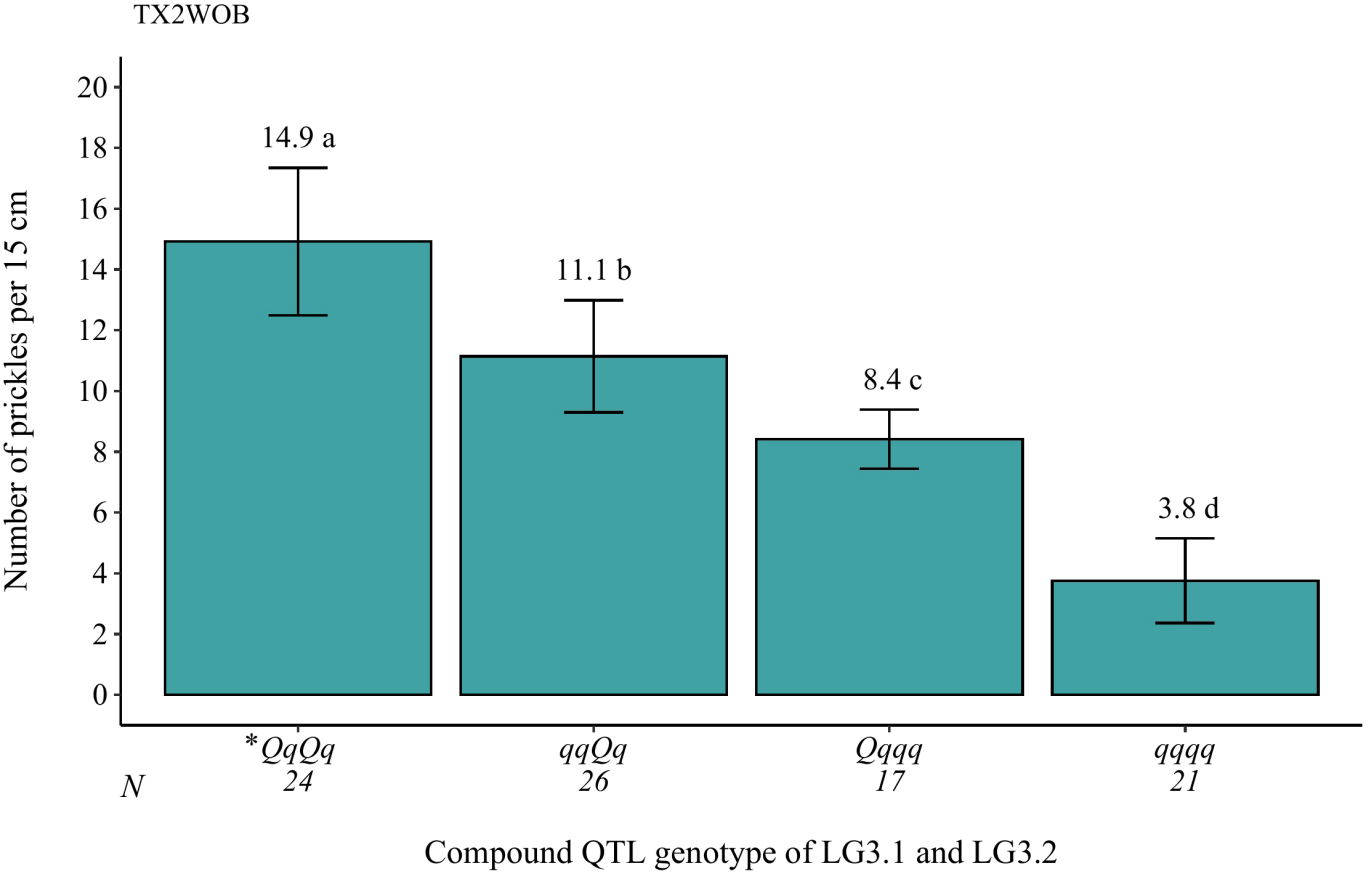
Analysis of the compound QTL genotypes from *q*SPCK.TX2WOB-LG3.1 and *q*SPCK.TX2WOB-LG3.2 for prickle density of the stem from all progenies in the TX2WOB population. Means not connected by the same letter are significantly different (p<0.05). *The first and second pairs of QTL alleles are for the LG3.1 and LG3.2 QTLs, respectively. N = Sample size.

The genotypes at qSPCK.TX2WOB-LG3.1 exhibited three distinct QTL genotypes (*qq*, *Qq*, *QQ*), with *q* and *Q* representing low and high SPCK, respectively. The average SPCK for offspring with *QQ*, *Qq*, and *qq* genotypes were 16.8, 11.6, and 6.9, respectively (**Figure 6A**). For *q*SPCK.TX2WOB-LG3.2, the average SPCK was 12.8 for *Qq* genotypes and 6.0 for *qq* genotypes; no individuals had the *QQ* genotype at this locus (**Figure 6B**). Generally, this population exhibited fewer unfavorable alleles (*Q*) related to increased SPCK than favorable alleles (*q*). Moreover, the interaction between these QTLs on LG3 was examined by analyzing their compound QTL genotypes. Individuals with two *Q*-alleles (heterozygous-*Qq* at both loci) displayed the highest SPCK, whereas those with four *q-*alleles had the lowest (**Figure 7**). The effect of *QQ*-genotypes could not be determined due to the absence of this QTL class at *q*SPCK.TX2WOB-LG3.2. The analysis showed that *q*SPCK.TX2WOB-LG3.2 had a greater impact on SPCK than *q*SPCK.TX2WOB-LG3.1, as a single *Q* dose at *q*SPCK.TX2WOB-LG3.2 increased SPCK more significantly than at *q*SPCK.TX2WOB-LG3.1.

As for TX2WSE, two QTLs on LG3 and one on LG6 were associated with SPCK, showing strong and positive evidence. The QTL *q*SPCK.TX2WSE-LG3.2 was located between 70.20 cM and 78.51 cM (42.50 Mbp–44.55 Mbp) with a peak at 72.26 cM, showing high intensity and a PVE of 49% (**Tables 1**, **2** and **Figure 5**). The other QTLs on LG3 and LG6 were considered minor, showing a PVE of 9%. Thus, *q*SPCK.TX2WSE-LG3.2 was considered a major QTL for this population. Three QTL genotype groups (*QQ*, *Qq*, and *qq*) were predicted at *q*SPCK.TX2WSE-LG3.2, with average SPCK values of 22.5, 13.5, and 4.1, respectively (**Figure 6C**). Similar to the TX2WOB, this population had a lower frequency of unfavorable alleles (*Q*) linked to increased SPCK than favorable alleles (*q*).

Regarding RPCK, a major QTL, *q*RPCK.TX2WOB-LG6 was mapped in TX2WOB between 63.1 cM and 67.0 cM (65.85 Mbp–66.05 Mbp) with a high intensity and a PVE of 17%. Another QTL with positive evidence was found on LG4, showing a PVE of 8% (**Tables 1**, **3** and **Figure 3**). In TX2WSE, three QTLs were identified on LG3 and LG6. The QTL *q*RPCK.TX2WSE-LG3, located between 14.79 cM and 18.39 cM (22.46 Mbp–27.80 Mbp), showed positive evidence and a PVE of 10%. Additionally, two QTLs on LG6 were identified with PVE up to 9% (**Tables 1**, **3** and **Figure 5**).

**Table 3:** QTL name, linkage group (LG), position of the nearest SNP marker to mode (peak), QTL interval, posterior intensity (QTL intensity), and phenotypic variance explained (PVE) for prickle density on the rachis (RPCK) phenotyped on TX2WOB (ten diploid rose families) and TX2WSE (six diploid rose families) populations in 2021 in Somerville, Texas.

QTL name	LG	Mode		Interval		QTL intensity	PVE (%)
		(cM)	(Mbp)	(cM)	(Mbp)		
*q*RPCK.TX2WOB-LG4	4	27.2	22.69	25.8	19.14	0.62	8
				29.2	28.63		
*q*RPCK.TX2WOB-LG6	6	65.0	65.52	63.1	65.85	1.16	17
				67.0	66.05		
*q*RPCK.TX2WSE-LG3	3	17.74	22.89	14.79	22.46	0.76	10
				18.39	27.80		
*q*RPCK.TX2WSE-LG6.1	6	52.68	51.82	48.34	50.88	0.53	8
				54.70	59.09		
*q*RPCK.TX2WSE-LG6.2	6	63.75	61.68	63.31	60.38	0.97	9
				66.63	61.91		

An inspection of genomic regions where QTLs were identified using the *Rosa chinensis* genome v1.0 assembly (Hibrand Saint-Oyant et al., 2018) revealed many candidate genes (**Supplementary Table 8**). These candidate genes were previously proposed to be linked with prickle development, including bHLH, C2H2 Zinc Finger, WD40 repeat, MYB, and WRKY families (Pattanaik et al., 2014; Ma et al., 2016; Huchelmann et al., 2017; Chopra et al., 2019) were also identified

### Haplotypes analysis, their effects, and sources

In this study, three major LG3 QTLs associated with the stem prickle density were considered for further analysis, namely, *q*SPCK.TX2WOB-LG3.1, *q*SPCK.TX2WOB-LG3.2, and *q*SPCK.TX2WSE-LG3.2. However, we extended haplotype analysis for the two overlapping minor QTLs between both traits related to the stem and rachis prickle (*q*SPCK.TX2WSE-LG3.1 and *q*RPCK.TX2WSE-LG3) and the LG6 QTL for rachis prickle (*q*RPCK.TX2WOB-LG6) that showed decisive evidence with high PVE.

### Stem prickle density

**Figure 8:**
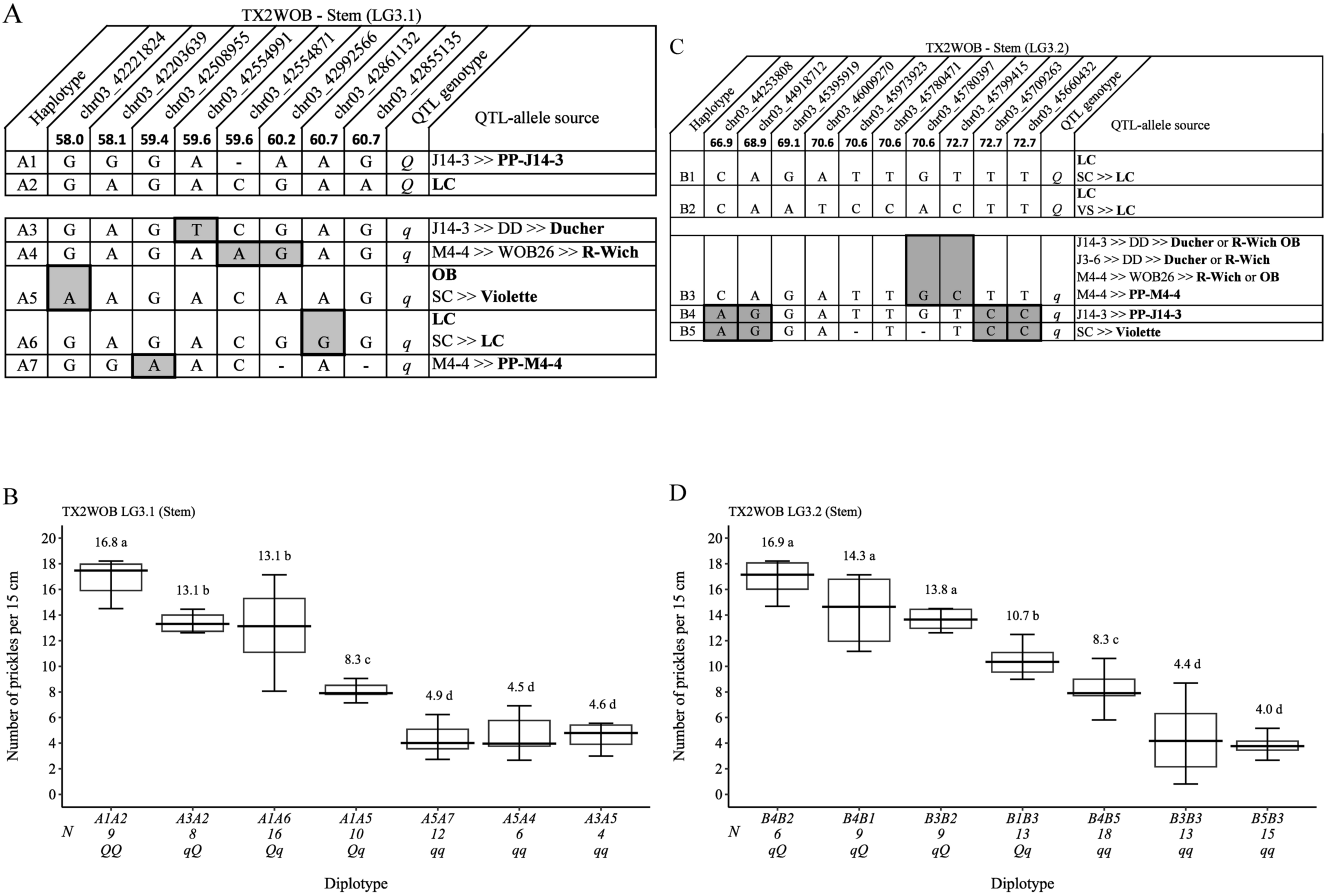
QTL genotypes, SNP haplotypes, alleles for predictive SNP markers associated with increasing (*Q*-alleles) or decreasing (*q*-alleles) stem prickle density, and sources **(A, C)**, and their diplotype effects **(B, D)** in diploid rose parents at the *q*SPCK.TX2WOB-LG3.1 and *q*SPCK.TX2WOB-LG3.2 loci, respectively. Predictive SNPs are shaded. Means not connected by the same letter are significantly different (*p<0.05*) QTL using the nonparametric multiple comparison Steel–Dwass test. N, diplotype sample size. LC, ‘Little Chief’; OB, ‘Old Blush’; SC, ‘Sweet Chariot’; R-Wich, *Rosa wichurana*.

Eight successive SNP markers within the QTL region of *q*SPCK.TX2WOB-LG3.1 (58.0 cM to 60.7 cM, 42.22 Mbp to 42.85 Mbp) (**Figure 8A**) spanning ~2.7 cM (~0.63 Mbp) were selected for haplotyping. Seven distinct SNP haplotypes were identified across eight parents. Haplotypes A1 and A2 were linked to increasing prickles on the stem and assigned to the *Q*-allele, and A3 to A7 were haplotypes related to decreasing prickles (*q*-allele). The estimation of diplotype effects indicated that A1(*Q*) had a larger effect than A3 (*q*) since the A1A2 and A1A5 diplotypes showed higher prickles than A3A2 and A3A5, respectively (**Figure 8B**). Haplotypes A4, A5, and A7 showed a similar magnitude in lowering prickles. The effect size of A2 (*Q*) was greater than A5 (*q*) when comparing A1A2 to A1A5 and A3A2 to A3A5. Likewise, A2 was greater than A6 (*q*) based on A1A2 to A1A6. Overall, A1A2 (*QQ*) showed the highest (~17), whereas A3A5, A5A7, and A5A4 (*qq*) had the lowest (~5) prickles (**Figure 8B**).

The pedigree map showed that the sources of A1 and A2 (*Q*-alleles) were PP-J14-3 and ‘LC’, respectively (**Figure 8A**). Also, haplotypes A3, A4, A6, and A7 were inherited from ‘Ducher’, ‘R-Wich’, ‘LC’, and PP-M4-4, respectively. In this study, the pedigree information revealed that some parents shared identical haplotypes and were inherited from various distinct sources. These haplotypes were considered identical-by-state (IBS), not identical-by-descent (IBD), such as A5 inherited from two sources, ‘OB’ and ‘Violette’. For *q*SPCK.TX2WOB-LG3.2, five unique haplotypes were defined with 10 SNPs spanning ~5.8 cM (~1.5 Mbp) across eight parents (**Figure 8C**). B1 and B2 were associated with increasing prickles on the stem and were assigned to the *Q*-allele, whereas B3, B4, and B5 were the haplotypes related to decreased prickles and assigned to the *q*-allele. The estimation of diplotype effects indicated that the effect of B1 and B2, which were associated with *Q*-alleles, could not be differentiated when comparing B4B2 and B4B1 diplotypes (**Figure 8D**). Likewise, B3, B4, and B5 had similar magnitudes in decreasing prickles when comparing B4B2 to B3B2 and B3B3 to B5B3 diplotypes. B1 and B2 appeared to lead to more prickles than B5 (*q*-allele) when comparing the diplotype B1B3 to B5B3 and B4B2 to B4B5, respectively. Also, the effect size of B1 was greater than B3 based on B1B3 and B3B3. Among diplotypes, B4B2 and B5B3 showed the highest (~17) and lowest (4) prickles at this locus (**Figure 8D**). The pedigree map showed that ‘LC’ was the only source *Q*-alleles of B1 and B2 (**Figure 8C**). Also, B4 and B5 came from PP-J14-3 and ‘Violette’, respectively. Lastly, B3 was inherited from four different sources ‘OB’, ‘R-Wich’, ‘Ducher’, or PP-M4-4.

**Figure 9:**
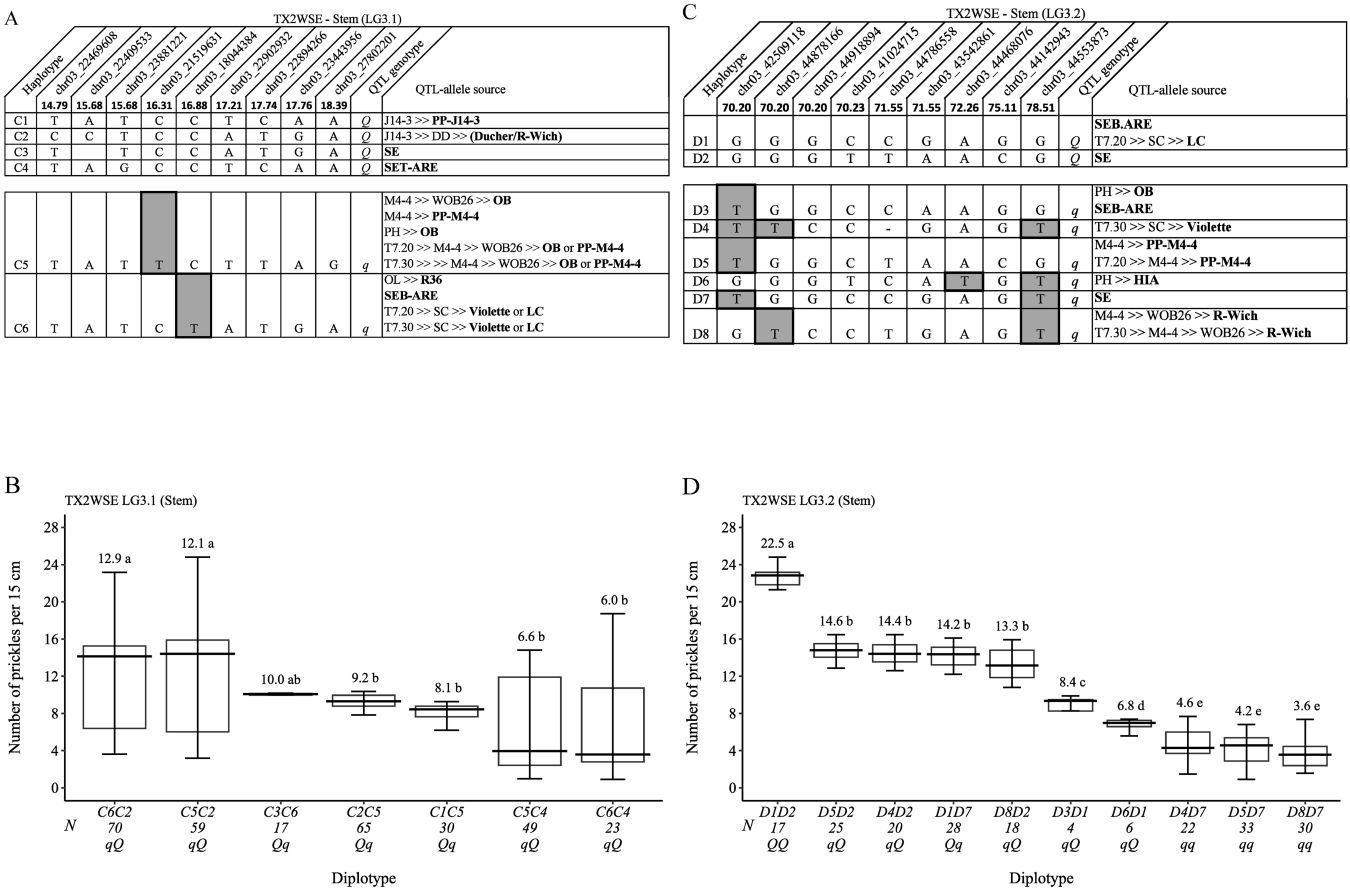
QTL genotypes, SNP haplotypes, alleles for predictive SNP markers associated with increasing (*Q*-alleles) or decreasing (*q*-alleles) stem prickle density, and sources **(A, C)**, and their diplotype effects **(B, D)** in diploid rose parents at the *q*SPCK.TX2WSE-LG3.1 and *q*SPCK.TX2WSE-LG3.2 loci, respectively. Predictive SNPs are shaded. Means not connected by the same letter are significantly different (*p<0.05*) QTL using the nonparametric multiple comparison Steel–Dwass test. N, diplotype sample size. T7.20, TAMU7-20; SC, ‘Sweet Chariot’; LC, ‘Little Chief’; SE, ‘Srdce Europy’; PH, ‘Papa Hemeray’; OB, ‘Old Blush’; T7.30, TAMU7-30; HIA, ‘Hiawatha’; R-Wich, *Rosa wichurana*.

Regarding TX2WSE and *q*SPCK.TX2WSE-LG3.1, six SNP haplotypes were identified using nine SNP markers (14.79 cM and 18.39 cM) spanning ~5.34 cM (~5.4 Mbp) (**Figure 9A**). Haplotypes C1 to C4 were associated with increasing prickles (*Q*-allele), and C5 and C6 were linked to decreasing prickles on the stem (*q*-allele). The haplotype results showed that C5 and C6 had similar effects in decreasing prickles when comparing C5C3 to C6C3 and C6C1 to C5C1. Due to the lack of diplotype combinations that are associated with *Q*-alleles, the effect of those haplotypes (C1, C2, C3, and C4) were not determined. However, diplotypes C6C1 and C5C1 showed the highest (greater than 12) and C6C3 showed the lowest (~6) prickles (**Figure 9B**). The pedigree records uncovered that *Q*-allele sources of C1, C3, and C4 were PP-J14-3, ‘SE’, and SET-ARE, respectively, whereas C2 came from a recombination event between DD parents (Ducher and R-wich) (**Figure 9A**). On the other hand, the *q*-allele sources of C5 were either ‘OB’ or PP-M4-4, and the C6 sources were ‘Violette’, ‘LC’, SEB-ARE, or R36.

Lastly, for *q*SPCK.TX2WSE-LG3.2, eight distinct SNP haplotypes were identified using nine SNP markers (70.20 cM and 78.51 cM) spanning ~8.30 cM (~2 Mbp) (**Figure 9C**). Haplotypes D3 to D8 were linked to decreasing prickles on the stem and assigned to *q*-alleles, whereas D1 and D2 were associated with increasing prickles (*Q*-allele). The analysis of diplotype showed that the effects of D5 and D4 could not be differentiated when comparing D5D2 to D4D2 and D4D7 to D5D7, and the same was observed between D4 and D8 when comparing D4D2 to D8D2 and D4D7 to D8D7 (**Figure 9D**). D2 (*Q*-allele) had a greater effect than D7 (*q*-allele) based on D1D2 to D1D7, D4D2 to D4D7, and D8D2 to D8D7 diplotypes. While D6 showed a smaller effect than D3 when comparing D3D1 to D6D1, D1 had a greater effect than D4 and D5 by comparing D1D7 to D4D7 and D1D2 to D5D2. In general, D1D2 and D8D7 showed the highest (~23) and lowest (~4) prickles, respectively (**Figure 9D**). The *Q*-allele source of D1 was SEB-ARE or ‘LC’, whereas D2 came from ‘SE’ (**Figure 9C**). The *q*-allele source of D3 was either ‘OB’ or SEB-ARE, D4 was ‘Violette’, D5 was PP-M4-4, D6 was HIA, D7 was ‘SE’, and D8 was ‘R-Wich’.

### Rachis prickle density

**Figure 10:**
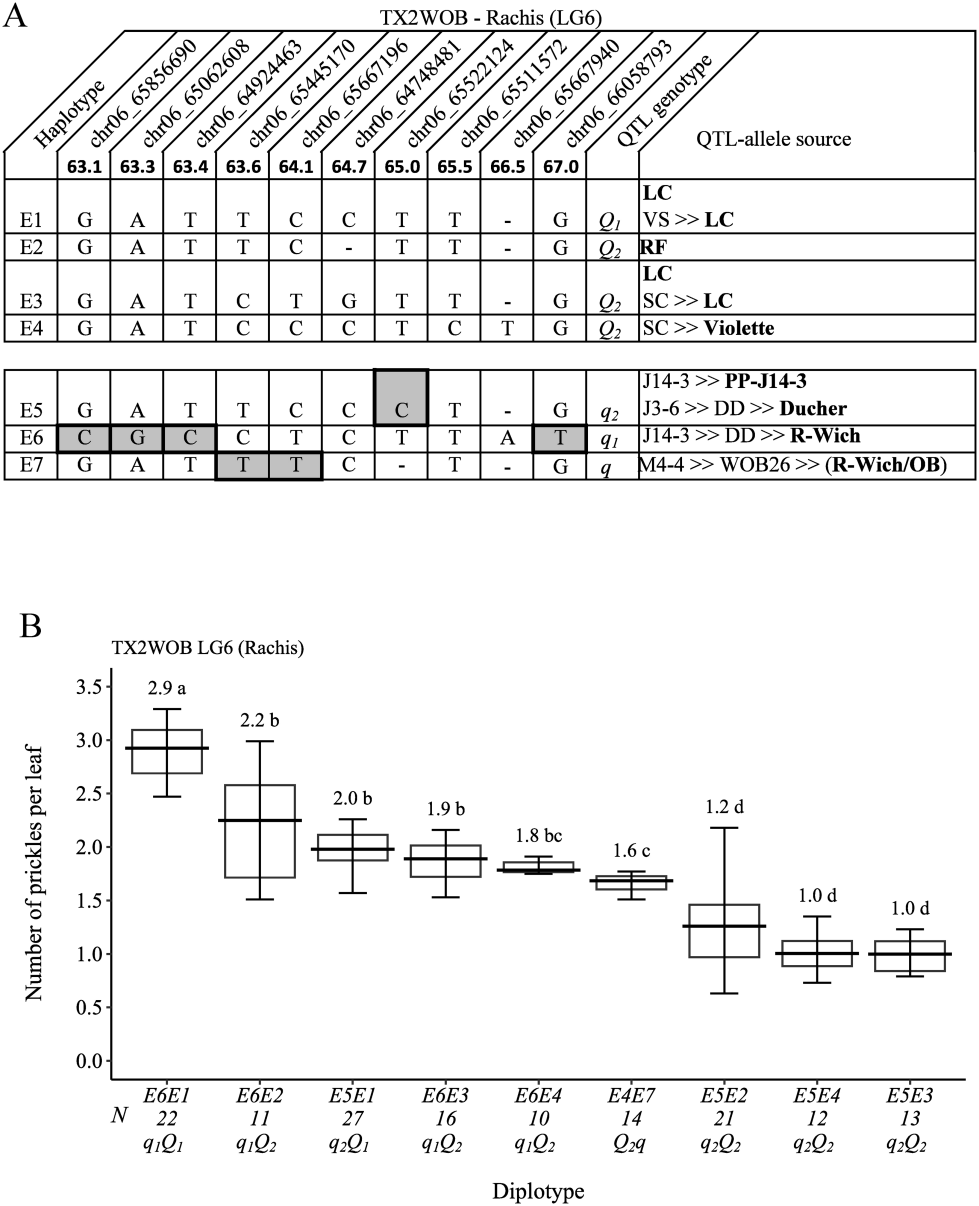
QTL genotypes for rachis prickle density in diploid rose parents with their SNP haplotypes, SNP sequences, sources, and alleles for predictive SNP markers associated with *Q-* or *q*-alleles for increasing or decreasing prickles, respectively. The predictive SNPs are shaded in **(A)**, and diplotype effects of the most common haplotypes associated with prickle density are shown in **(B)** at the *q*RPCK.TX2WOB-LG6 locus. Multi-allelic series *Q*- and *q*-alleles are assigned subscript index numbers. Means not connected by the same letter are significantly different (*p<0.05*) using the non-parametric multiple comparison Steel–Dwass test. The *q* without a subscript indicates cases where the haplotype could not be categorized due to the lack of appropriate diplotype combination. N, diplotype sample size. LC, ‘Little Chief’; SC, ‘Sweet Chariot’; VS, ‘Vineyard Song’; R-Wich, *Rosa wichurana*; OB, ‘Old Blush’.

A total of 10 SNPs were in the *q*RPCK.TX2WOB-LG6 region (63.1 cM and 67.0 cM) spanning ~3.9 cM (~0.2 Mbp) (**Figure 10A**) chosen for haplotyping, which revealed seven SNP haplotypes. Haplotypes E1 to E4 were associated with increasing rachis prickles (*Q*-allele), whereas E5, E6, and E7 were related to decreasing prickles (*q*-allele), and E5 and E6 were the most common haplotypes. The estimation of diplotype effects showed that E1 (*Q*-allele) had a greater effect than E2, E3, and E4 (*Q*-alleles), in which the last haplotypes had a similar magnitude when comparing E6E1, E6E2, E6E3, and E6E4 and E5E1, E5E2, E5E4, E5E3 (**Figure 10B**). Also, E5 had more effect in decreasing prickles than E6 (E6E1 to E5E1, E6E2 to E5E2, and E6E3 to E5E3). Hence, multiple QTL alleles of different effects were found at this locus. The haplotype effects order was E1 > [E2 = E3 = E4] > [E6] > [E5] corresponding to *Q_1_*, *Q_2_*, *q_1_*, and *q_2_*, respectively. However, the underrepresentation of diplotype combinations associated with E7 hinders our ability to determine its magnitude on decreasing prickles compared with others. The highest (~3) and lowest (~1) prickle densities were seen in E6E1 (*q_1_Q_1_*) and E5E3 (*q_2_Q_2_*), respectively (**Figure 10B**). ‘LC’ was the source *Q*-alleles of E1 and E3, and ‘RF’ and ‘Violette’ were the source *Q*-alleles of E2 and E4, respectively (**Figure 10A**). E5 (*q*-allele) was inherited from three distinct sources PP-J14-3 ‘OB’, or ‘Ducher’. E6 and E7 (*q*-alleles) came from ‘R-Wich’ and a recombination event between WOB26 parents (‘R-wich’ and ‘OB’), respectively.

**Figure 11:**
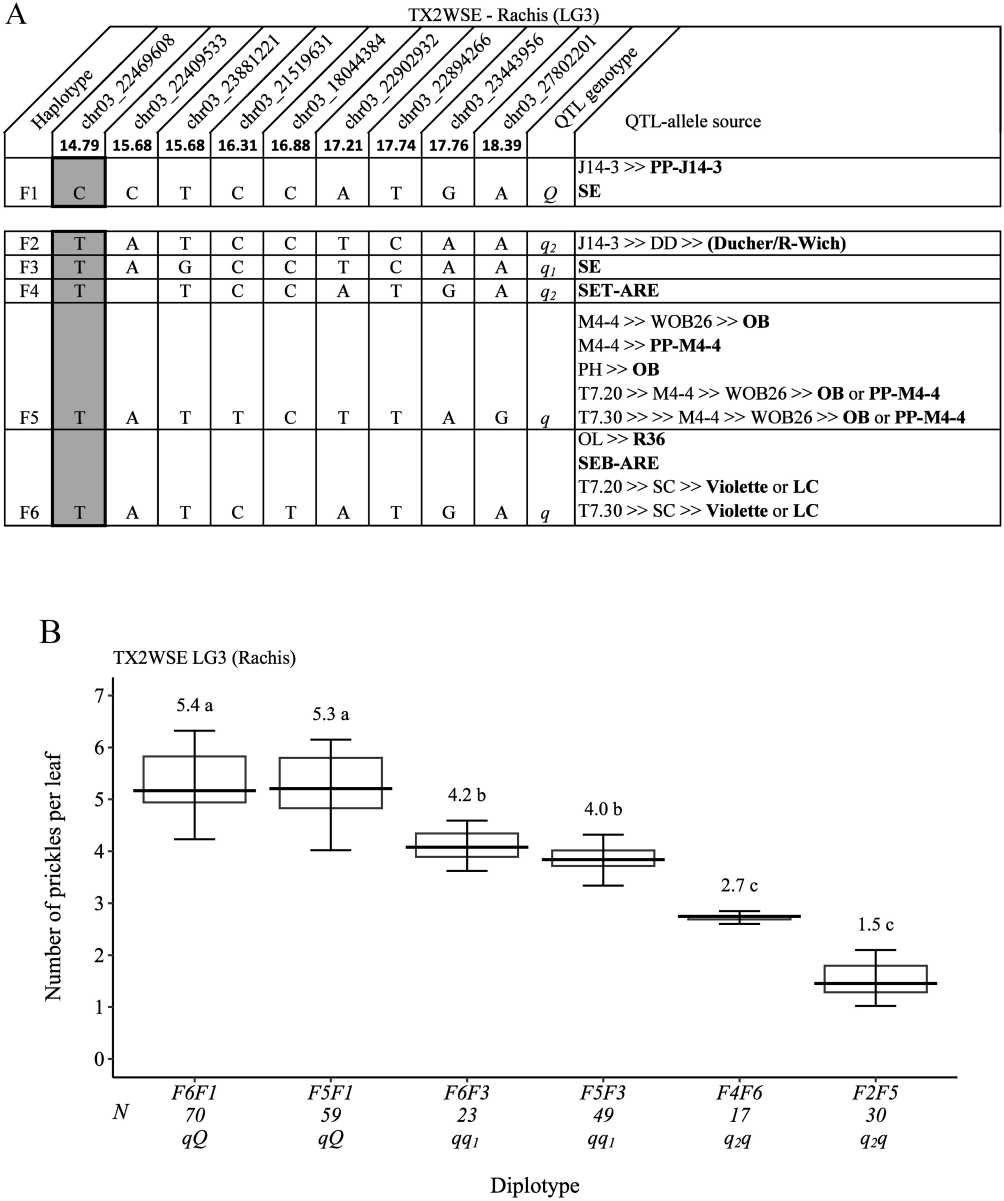
QTL genotypes for rachis prickle density in diploid rose parents with their SNP haplotypes, SNP sequences, sources, and alleles for predictive SNP markers associated with *Q-* or *q*-alleles for increasing or decreasing prickles, respectively. The predictive SNPs are shaded in **(A)**, and diplotype effects of the most common haplotypes associated with prickle density are shown in **(B)** at the *q*RPCK.TX2WSE-LG3 locus. Multi-allelic series *q*-alleles are assigned subscript index numbers. Means not connected by the same letter are significantly different (*p<0.05*) using the non-parametric multiple comparison Steel–Dwass test. The *q* without a subscript indicates cases where the haplotype could not be categorized due to the lack of appropriate diplotype combination. N, diplotype sample size. T7.20, TAMU7-20; SC, ‘Sweet Chariot’; LC, ‘Little Chief’; SE, ‘Srdce Europy’; PH, ‘Papa Hemeray’; OB, ‘Old Blush’; T7.30, TAMU7-30; R-Wich, *Rosa wichurana*.

For TX2WSE, nine SNP markers in the QTL region of *q*RPCK.TX2WSE-LG3 (14.79 cM and 18.39 cM) spanning ~3.6 cM (~5.4 Mbp) (**Figure 11A**) were selected for haplotype analysis. Six SNP haplotypes were identified in which five haplotypes from F2 to F6 were linked to decreasing prickles on the rachis (*q*-allele), and F1 was the only haplotype associated with increasing prickles (*Q*-allele). The estimation of diplotype effects revealed that F5 (*q*) and F6 had an equal effect in decreasing prickle (F6F1 to F5F1 and F6F3 to F5F3) (**Figure 11B**). Also, the results showed that haplotypes F2 (*q_2_*) and F4 (*q_2_*) appeared to have more effect in decreasing prickles than F3 (*q_1_*). Generally, F6F1 and F5F1 had the highest (~5), and F2F5 had the lowest (~2) prickles (**Figure 11B**).

Also, the pedigree map showed that the *Q*-allele source of F1 was PP-J14-3, whereas the *q*-allele source of F2 was J14-3, which arose from recombination events between the parents of DD (‘Ducher’ and ‘R-wich’) (**Figure 11A**). Also, F3 and F4 were inherited from ‘SE’ and SET-ARE, respectively. F5 came from ‘OB’ or PP-M4-4, whereas F6 was inherited from either ‘Violette’, ‘LC’, SEB-ARE, or R36.

## Discussion

### Heritability and correlation

In this study, SPCK exhibited a moderately high h^2^ (0.60 to 0.64) and a high (0.83) H^2^. A high h^2^ (0.97) was previously reported for SPCK using diploid rose germplasm (Zhou et al., 2020). Thus, the findings in this and earlier research with diploid and tetraploid roses imply that environmental effects have little effect on SPCK (Gitonga et al., 2014). In contrast, the leaf rachis prickles showed low to moderate h^2^ (0.25 to 0.42) and moderate H^2^ (0.40). Gitonga et al. (2014) described the stem and rachis prickles as having high H^2^ and low G×E interaction, indicating that these traits would be suitable for further genetic studies. The variability in heritability was not surprising, considering that heritability is known to be population-specific and influenced by factors such as experimental design, statistical method, and others (Visscher et al., 2008). Overall, this study highlighted the important additive effects of these traits. The weak correlation between stem and rachis prickles seen in this and other studies (Rajapakse et al., 2001) as well as the observation that the genes responsible for regulating these traits are located on distinct LGs, indicate that these traits segregate independently.

### QTL mapping

#### Stem prickles

In both populations, four and three QTLs were mapped for TX2WOB and TX2WSE, respectively, and distributed on LG2 (only TX2WOB), LG3, and LG6. This finding implies that prickle density is a polygenic trait, confirming the hypothesis that multiple loci are responsible for this trait (Crespel et al., 2002; Koning-Boucoiran et al., 2012; Gitonga et al., 2014; Bourke et al., 2018; Hibrand Saint-Oyant et al., 2018; Zhou et al., 2020; Zhong et al., 2021). Among these detected QTLs, the two neighboring QTLs (*q*SPCK.TX2WOB-LG3.1 and *q*SPCK.TX2WOB-LG3.2) mapped on the middle part of LG3 (42.22 Mbp to 42.85 Mbp and 44.25 Mbp to 45.66 Mbp, PVE18 to 43%) in TX2WOB overlapped with the single QTL *q*SPCK.TX2WSE-LG3.2 (42.50 Mbp to 44.55 Mbp, PVE 49%) of TX2WSE. This finding coincided with two previous reports that used populations derived from genetic backgrounds similar to those in the present study. Hibrand Saint-Oyant et al. (2018) identified a large QTL (or two neighboring QTLs) located between 31.0 Mbp to 46.5 Mbp on LG3 associated with stem prickle density. Zhou et al. (2020) also consistently detected two overlapping QTLs on LG3 spanning the regions between 36.51 Mbp to 46.44 Mbp and 41.64 Mbp to 42.31 Mbp, with the closest markers at peaks of 44.45 Mbp and 42.31 Mbp.

Interestingly, the qualitative analysis for stem prickles revealed that the chromosomal region from 43.70 Mbp to 46.66 Mbp on LG3 also controlled the presence/absence of prickles (**Figures 3** and **5**). Likewise, Zhou et al. (2020) reported that the closest markers associated with the presence of prickles were at 40.21 Mbp and 42.31 Mbp on LG3 and co-localized with QTL intervals involved in regulating prickle density in diploid roses.

The LG3 QTL reported in both diploid and tetraploid populations by multiple studies (Crespel et al., 2002; Linde et al., 2006; Koning-Boucoiran et al., 2012; Bourke et al., 2018; Hibrand Saint-Oyant et al., 2018). Hence, this study confirms that the LG3 QTL between ~42.0 Mbp to 45.0 Mbp is robust and independent of the genetic background and environment. Also, the two QTLs on LG6 of both populations overlapped with the previously mapped QTL on LG6 (~1.33 Mbp to 64.12 Mbp, peak at ~5.41 Mbp) (Zhou et al., 2020), which was close to the peak of *q*SPCK.TX2WSE-LG6 (7.12 Mbp). The LG2 QTL was novel and specific to TX2WOB.

Regarding rachis prickle, there were five QTLs mapped across both populations, with PVE ranging from 8% to 17%, and no QTL overlapped over populations. These were mapped to LG3 (*q*RPCK.TX2WSE-LG3), LG4 (*q*RPCK.TX2WOB-LG4), and LG6 (*q*RPCK.TX2WSE-LG6.1, *q*RPCK.TX2WSE-LG6.2, *q*RPCK.TX2WOB-LG6). *q*RPCK.TX2WSE-LG3 co-localized with *q*SPCK.TX2WSE-LG3.1 and *q*RPCK.TX2WOB-LG4 was ~1.4 Mbp upstream of the previously identified QTL on LG4 for stem prickles between 30.43 Mbp and 56.10 Mbp (Zhou et al., 2020). The QTLs on LG6 were discovered at three different chromosomal segments. Two neighboring QTLs were mapped in TX2WSE (*q*RPCK.TX2WSE-LG6.1 and *q*RPCK.TX2WSE-LG6.2) and overlapped with *q*SPCK.TX2WOB-LG6. A third QTL on LG6 (*q*RPCK.TX2WOB-LG6) was mapped in TX2WOB at the distal part of the LG and downstream of the first two QTLs. All these QTLs overlapped with the QTL earlier reported for the stem prickles (Zhou et al., 2020).

The co-localization between QTLs of SPCK and RPCK found in this study has not been reported. This may indicate these minor loci on LG3 (22.46 Mbp to 27.80 Mbp) and LG6 (50.88 Mbp to 61.91 Mbp) are responsible for prickle density in both traits. Bourke et al. (2018) reported that stem and rachis prickles are regulated differently as the major QTLs for both traits mapped at different LGs, namely, LG3 and LG4, respectively. Thus, this study mapped several QTLs, suggesting that multiple genes influence stem and rachis prickles. The LG3 QTL between ~42.0 Mbp to 45.0 Mbp for stem prickles was consistent across population and year. Minor QTLs on LG3 (TX2WSE) and LG6 (both populations) were associated with both traits, whereas others were environment-specific. The PBA approach enabled discovering new QTLs and validating the major LG3 QTL for stem prickles. Further mapping with diverse germplasm is essential for a comprehensive understanding. Increasing population size can also enhance QTL effect accuracy.

### Haplotype characterization of QTLs

Haplotype analyses for the QTLs on LG3 for stem prickle density for both populations and LG6 and LG3 QTLs detected for the rachis prickle for TX2WOB and TX2WSE, respectively, uncovered SNP haplotypes and predictive SNP markers related to increasing/decreasing QTL alleles. For the stem prickle QTLs, this analysis identified seven, five, six, and eight unique haplotypes for *q*SPCK.TX2WOB-LG3.1, *q*SPCK.TX2WOB-LG3.2, *q*SPCK.TX2WSE-LG3.1, and *q*SPCK.TX2WSE-LG3.2, respectively.

Seven and six distinct haplotypes were revealed for rachis prickle density QTL *q*RPCK.TX2WOB-LG6 and *q*RPCK.TX2WSE-LG3. The haplotype results also revealed that only haplotypes C5 and C6 of the minor LG3 QTL for the stem prickle density (*q*SPCK.TX2WSE-LG3.1) and F5 and F6 of the rachis prickle density (*q*RPCK.TX2WSE-LG3) were in the coupling phase. Interestingly, genes controlling these traits were reported previously and are located on different chromosomal regions (Rajapakse et al., 2001; Bourke et al., 2018).

For breeding selection, based on the pedigree records, the ancestors ‘OB’, PP-M4-4, and ‘R-Wich’ were common sources of *q*-alleles across populations, and ‘LC’ and ‘SE’ were the source of *Q*-alleles in the TX2WOB and TX2WSE populations, respectively. In this study, identifying the parents of breeding line M4-4 and ‘R-Wich’ as sources of the *q*-allele was expected as both are prickle-free roses. Also, selection for a specific pair of haplotypes (diplotype) across three loci (two loci for the stem and a single locus for the rachis prickle density) for each studied population could be used to identify and select prickle-free roses. For instance, diplotype combinations of A5A7, B5B3, and either E4E7 or E3E7 in SC×M4-4 progeny were prickle-free roses in TX2WOB (**Supplementary Table 6**). Moreover, a combination of C5C3, D5D7, and F5F3 (M4-4×SE), C5C1, D5D7, and F5F1 (T7.20×SE), or C6C3, D8D7, and F6F3 (T7.30×SE) diplotypes could be used to identify prickle-free roses in TX2WSE (**Supplementary Table 7**).

Overall, the findings of this study will facilitate breeders in parental/seedling selection to develop new rose cultivars with no or few prickles specifically for cut rose cultivars, as they are in high demand by producers and breeders. Ultimately, the major QTL on LG3 (~42.0 Mbp to 45.0 Mbp) is reliable and could be employed to develop high-throughput DNA tests for routine use in a DNA-informed breeding program (Demirel et al., 2023; da Silva Linge et al., 2024). Additional QTL mapping through PBA for rose prickles using different and wider genetic materials is necessary to validate minor QTLs and clarify the relationship between rachis and stem prickles.

### Candidate genes within mapped QTL intervals

The knowledge of the genetic regulatory networks underlying prickle morphogenesis in roses is poor. However, Hibrand Saint-Oyant et al. (2018) found that a WRKY transcription factor (TF), RcTTG2, at 33.40 Mbp on LG3, was close to the major QTL affecting stem prickles. This gene was ~9.0 Mbp upstream of *q*SPCK.TX2WOB-LG3.1 and *q*SPCK.TX2WSE-LG3.2, and ~11.0 Mbp of *q*SPCK.TX2WOB-LG3.2. Since rose prickles may originate from a trichome-like structure, several rose homologs of TFs involved in trichome initiation and development have been reported in *Rosa chinensis* (Zhou et al., 2020). QTLs in this study harbored potential candidate genes, including bHLH, C2H2 Zinc Finger, WD40 repeat, MYB, and WRKY families (Pattanaik et al., 2014; Ma et al., 2016; Huchelmann et al., 2017; Chopra et al., 2019) (**Supplementary Table 8**).

Regarding the bHLH family, RC6G0407800 (a TT8 homolog) is located in the *q*RPCK.TX2WSE-LG6.1 region has been reported for its significant involvement in the development of marginal trichomes (Zhou et al., 2020). Several C2H2 TFs were identified in the LG3 and LG6 QTL regions. RC3G0150000 at 22.33 Mbp overlapped with LG3 QTLs is closely related to GLABROUS INFLORESCENCE STEMS proteins (GIS2) regulating trichome formation on inflorescence stems. Multiple WD40 TFs were located within all mapped QTLs in this study. However, they were mainly clustered within QTLs at 42.0 Mbp–44.0 Mbp on LG3, 20.0 Mbp–28.0 Mbp on LG4, and 51.0 Mbp–61.0 Mbp on LG6. RC3G0186600 at 27.14 Mbp on LG3 belongs to the same clade as RC1G0586100 and shows solid similarity to TRANSPARENT TESTA GLABRA 1(TTG1), and was reported to be involved in trichome and root hair development (Zhou et al., 2020).

Two copies of the WRKY family TFs were identified in the QTL intervals on LG3 and LG6. One of the TFs was located at 44.80 Mbp on LG3 and seemed to be closer to WRKY74, as previously revealed by Hibrand Saint-Oyant et al. (2018). The WRKY family has diverse plant biological functions, including biotic and abiotic stress responses, seed and trichome development, senescence, and embryogenesis (Bakshi and Oelmüller, 2014). A MYB TF family protein was identified at 65.91 Mbp on LG6 to play important roles in several biological processes in plants, and trichome initiation and development is one of them (Pattanaik et al., 2014), as mentioned above.

However, a recent study involving glabrous and non-glandular individuals, derived from ‘R-Wich’ and ‘OB’ cross, employed transcriptomic analysis during stages of prickle development in roses. The findings revealed that candidate genes commonly linked to trichome initiation and development played no role in prickle development (Zhou, 2021). They found 43 significant differentially expressed genes that might be good candidates for prickle initiation and development in roses (Zhou, 2021). Interestingly, 11 of these potential genes were clustered within the genomic region between 42 Mbp to 45 Mbp on chromosome 3, where the major QTL affecting stem prickles was detected in the present study. These genes were involved in auxin biosynthesis (RC3G0359600), preimplantation development (RC3G0373700), defense response (RC3G0353900, RC3G0384500, RC3G0385400, RC3G0402100, and RC3G0380200), and organ development (RC3G0356400, RC3G0386900, RC3G0389900, RC3G0351000). Also, the study highlighted a RC3G0386900, RC3G0359600, and RC3G0389900 as among the best candidate genes in prickle initiation based on their potential functions (Zhou, 2021).

RC3G0359600 at 42.72 Mbp on chromosome 3, which encoded an amidase signature homolog protein, is involved in the auxin and indoleacetic acid biosynthesis (Pollmann et al., 2003). The gene exhibited significant expression throughout various stages of prickle development in samples of the prickly rose, whereas its expression was notably suppressed in samples lacking prickles. This observation suggests that auxin positively regulates the initiation and progression of prickle formation, as suggested by Zhou (2021). RC3G0386900 at 44.26 Mbp on chromosome 3 belonged to NAD(P)-linked oxidoreductase superfamily protein and was found to be highly expressed in the early stage of glabrous stems and reported to be a good candidate for repressing prickle initiation (Zhou, 2021). RC3G0389900 at 44.62 Mbp on chromosome 3 belonged to a WUSCHEL-related homeobox 1 (WOX) gene family and is known to be involved in organ development in plants, including shoot meristem (Kieffer et al., 2006), root apical meristem (Sarkar et al., 2007), ovule development (Park and Luger, 2006), petal and carpel fusion (Vandenbussche et al., 2009), lateral root development and root hair formation (Sun et al., 2017), and repressing prickle initiation in roses (Zhou, 2021).

Likewise, all mapped QTL regions harbored numerous candidate genes that were reported earlier as involved in prickle development in *Rosa* (Zhou, 2021) and *Solanum melongena* (Zhang et al., 2021). For instance, candidate genes encoded key proteins related to DNA replication, cell division, cell cycle/proliferation, cell wall modification, cell wall organization, macromolecule metabolic processes, auxin, ethylene, and salicylic acid response.

As the two multi-parental populations used in this study have a genetic background of ‘Basye’s Thornless’ (BT), a prickle-free cultivar of *R. wichurana*, we observed that most of the candidate genes mentioned above in *Rosa chinensis* ‘OB’ genome (Hibrand Saint-Oyant et al., 2018) were also present in the BT genome (Zhong et al., 2021). This finding aligns with Zhong et al. (2021), who identified the major QTL on LG3 affecting stem prickles between 13.97 Mbp and 16.23 Mbp in the BT genome (which corresponds to ~29.04 Mbp to 31.66 Mbp in the *Rosa chinensis*) encompasses 117 homologous genes shared between the BT and *Rosa chinensis* genomes.

In general, other studies and ours suggest various plausible gene candidates behind discovered QTL, but additional work is needed to validate these candidates and decipher the gene network that controls prickle development.

## Conclusion

The PBA approach was successfully used on two multi-parental populations to demonstrate that the LG3 QTL is the major QTL that controls stem prickle density, with additional minor QTLs for both traits. The major QTL on LG3, between 42.22% and 45.66 Mbp is associated with the stem prickle density and the presence/absence of prickles simultaneously. There is co-localization between minor QTLs for stem and rachis prickles. Favorable alleles (*q*) associated with decreasing prickle density were traced back to ‘R-Wich’, ‘OB’, and PP-M4-4, whereas unfavorable alleles (*Q*) came from ‘LC’ in TX2WOB and ‘SE’ in TX2WSE. These findings will guide parental selection to improve rose aesthetic quality regarding prickle density. They also pave the way for DNA-informed techniques to develop roses with no or minimal prickles, aligning with consumer and breeder preferences, and conserving resources.

## Data Availability

The data presented in the study are deposited in the “https://www.rosaceae.org/” Genome Database for Rosaceae repository, accession number “https://www.rosaceae.org/search/node/tfGDR1075” tfGDR1075.
