# Supplementary figures and images for: Genetic dissection of stem and leaf rachis prickles in diploid rose using a pedigree-based QTL analysis
Source: Front Plant Sci. 2024 Sep 18;15:1356750. doi: 10.3389/fpls.2024.1356750 (PMC11445041; doi:10.3389/fpls.2024.1356750)

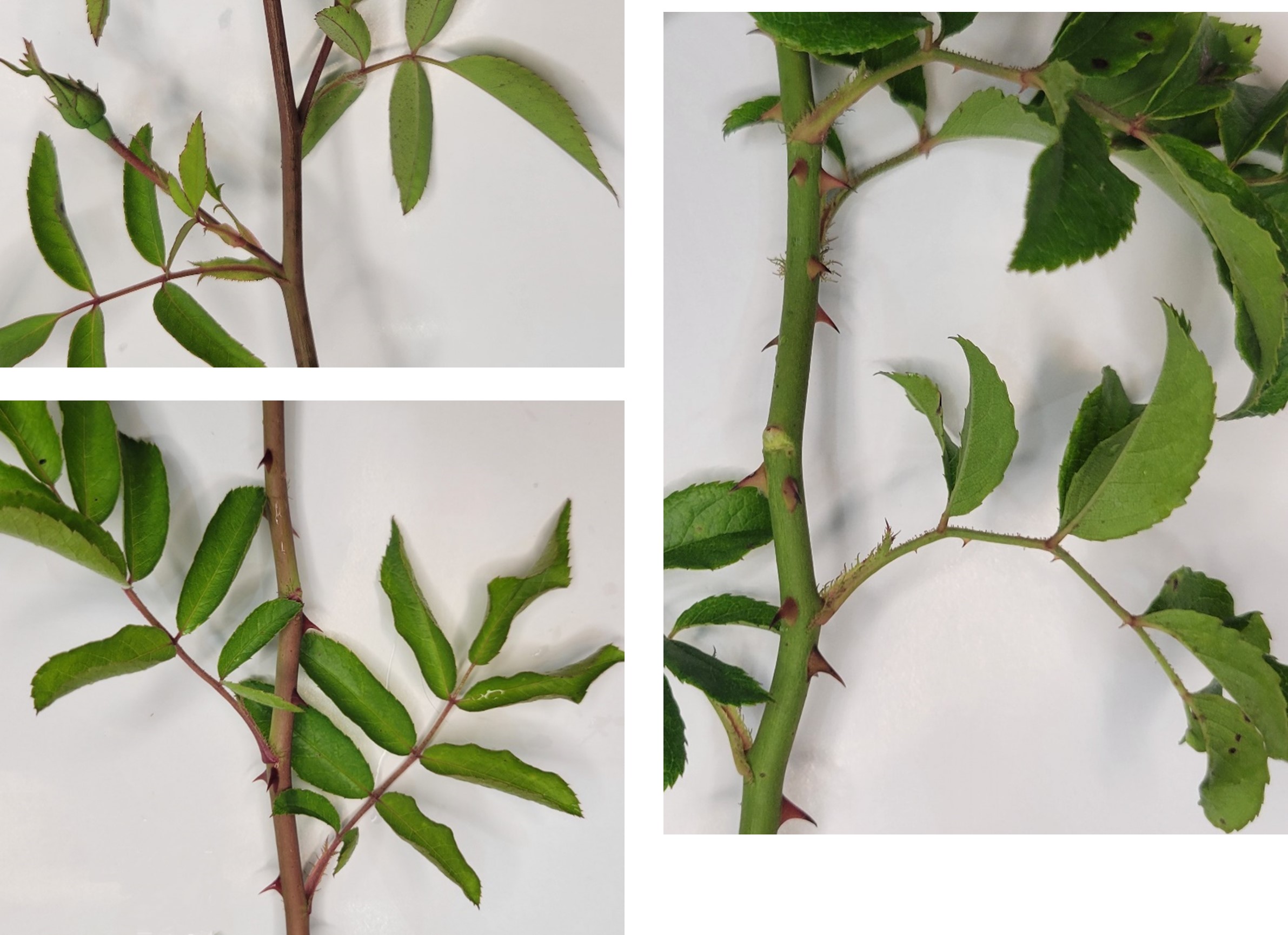

Supplement: Supplementary file 1 [file Image1.jpeg]

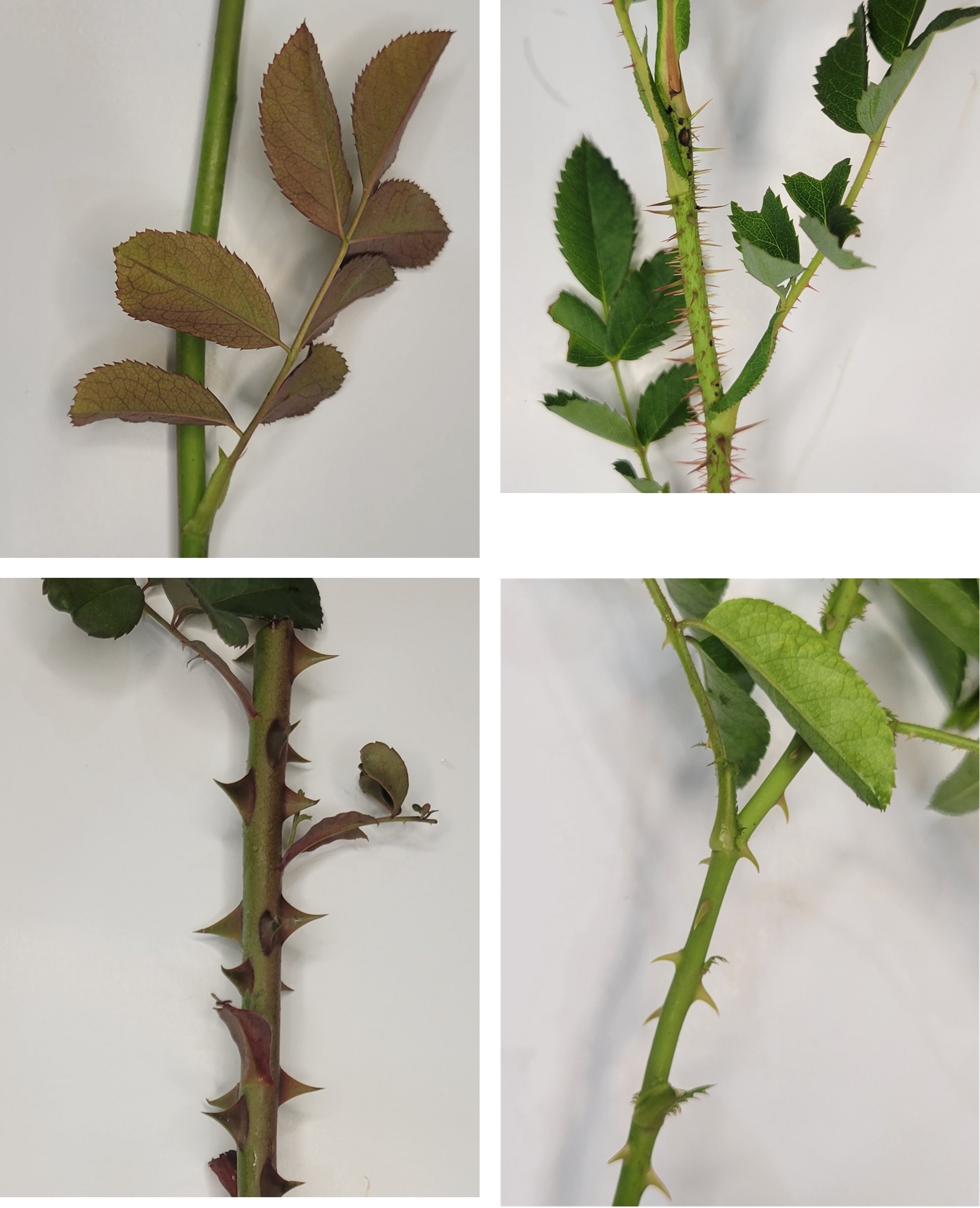

Supplement: Supplementary file 2 [file Image2.jpeg]

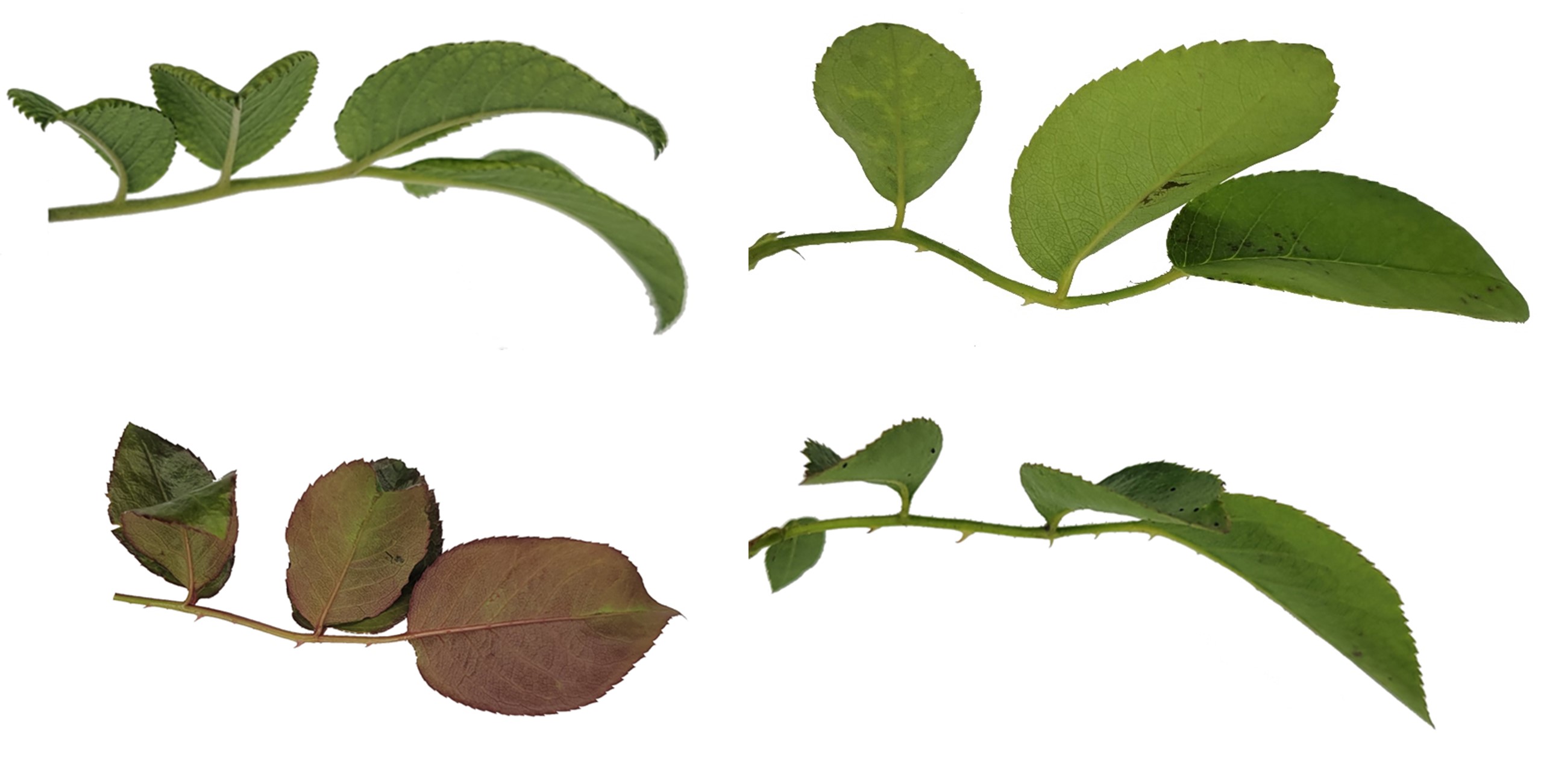

Supplement: Supplementary file 3 [file Image3.jpeg]

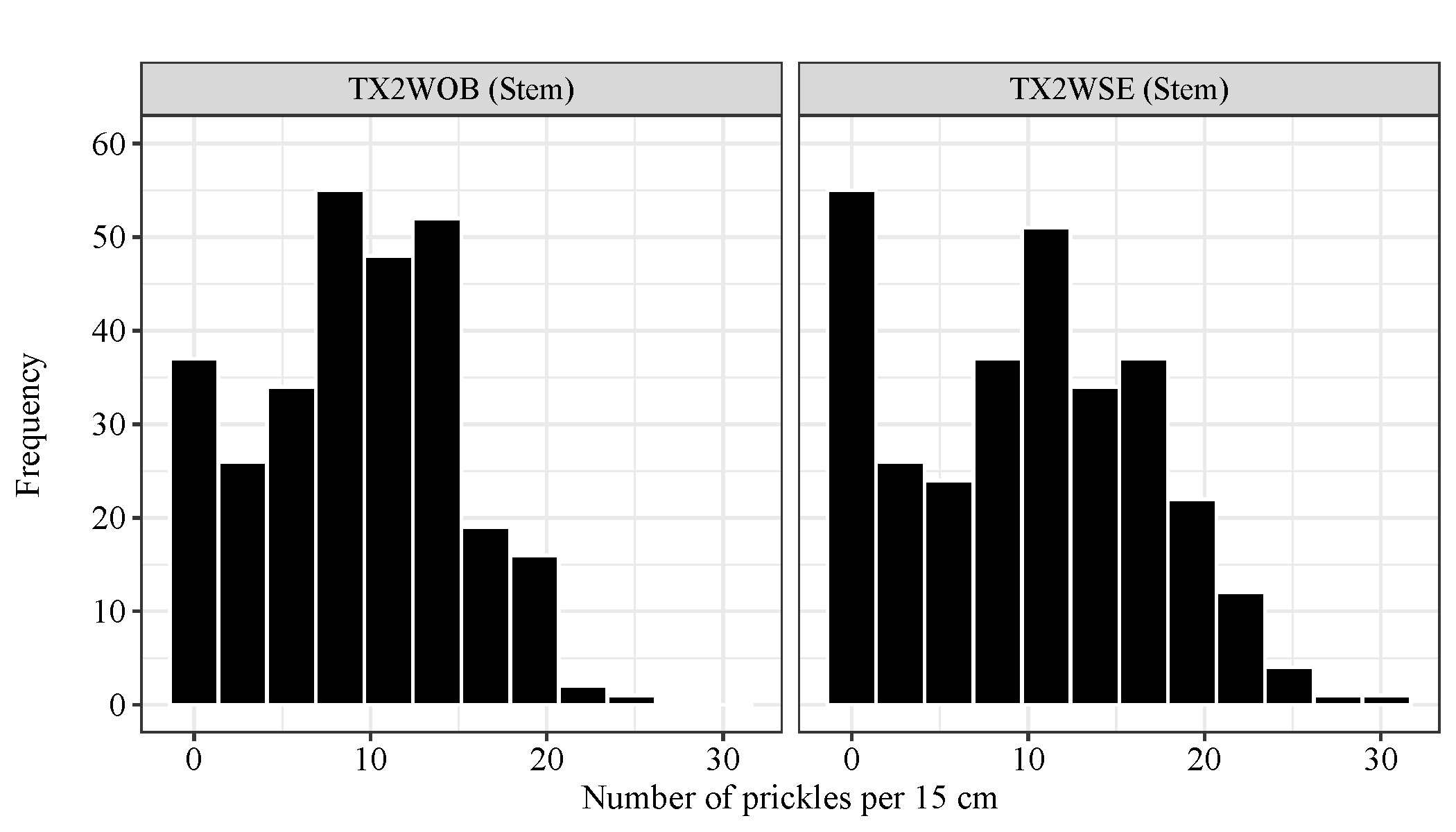

Supplement: Supplementary file 4 [file Image4.jpeg]

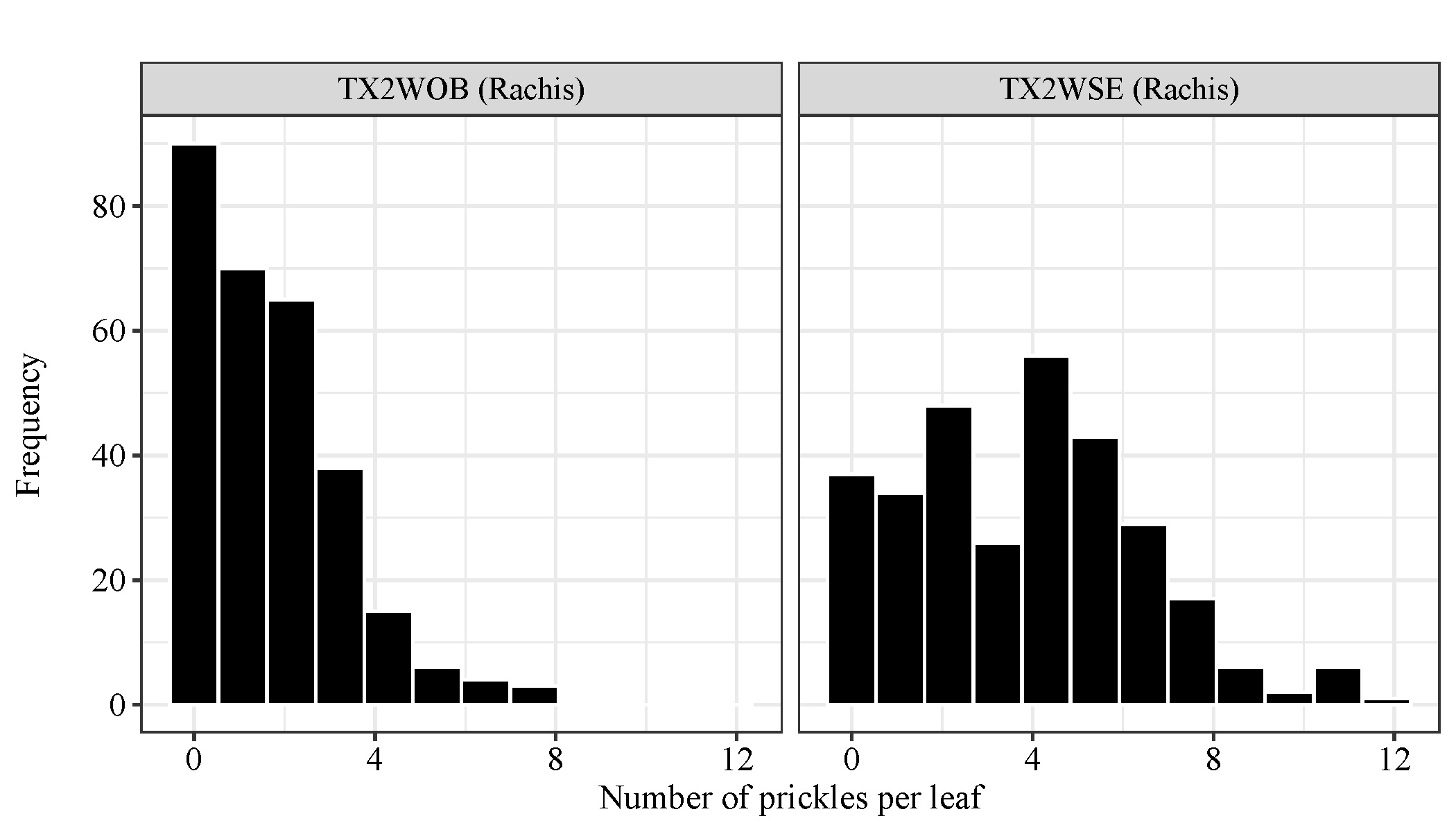

Supplement: Supplementary file 5 [file Image5.jpeg]
