# Supplementary material for: Genetic dissection of stem and leaf rachis prickles in diploid rose using a pedigree-based QTL analysis
Source: Front Plant Sci. 2024 Sep 18;15:1356750. doi: 10.3389/fpls.2024.1356750 (PMC11445041; doi:10.3389/fpls.2024.1356750)
Supplement: Supplementary file 6 [file Table1.docx]

Supplementary Tables

| **Supplementary Table 1**. Parentage and number of progenies for diploid rose multi-parental populations used for QTL analysis in Somerville, Texas in 2021 for TX2WOB and TX2WSE. | | | | | | | | |
| --- | --- | --- | --- | --- | --- | --- | --- | --- |
| TX2WOB | | | |  | TX2WSE | | | |
| Family | Female Parent | Male Parent | 2021 |  | Family | Female Parent | Male Parent | 2021 |
| J14-3×LC | J06-20-14-3 | Little Chief | 31 |  | J14-3×PH | J06-20-14-3 | Papa Hemeray | 118 |
| J14-3×RF | J06-28-4-6 | Red Fairy | 34 |  | M4-4×SEimp | M4-4 | Srdce Europy | 32 |
| J14-3×SC | J06-20-14-3 | Sweet Chariot | 40 |  | PH×SEB-ARE | Papa Hemeray | Swamp Rose EB-ARE | 10 |
| J14-3×VS | J06-20-14-3 | Vineyard Song | 49 |  | SET-ARE×OL | *R. setigera*-ARE | Ole | 21 |
| M4-4×SC | M4-4 | Sweet Chariot | 5 |  | T7-20×SEimp | TAMU7-20 | Srdce Europy | 93 |
| OB×J3-6 | Old Blush | J06-30-3-6 | 43 |  | T7-30×SEimp | TAMU7-30 | Srdce Europy | 80 |
| OB×M4-4 | Old Blush | M4-4 | 8 |  |  |  |  |  |
| OB×RF | Old Blush | Red Fairy | 43 |  |  |  |  |  |
| SC×J14-3 | Sweet Chariot | J06-20-14-3 | 21 |  |  |  |  |  |
| SC×M4-4 | Sweet Chariot | M4-4 | 24 |  |  |  |  |  |
| Total |  |  | 298 |  |  |  |  | 355 |

| **Supplementary Table 2**. Statistical summary of the five individual diploid rose maps and the integrated consensus map (TX2WOB ICM) by linkage group (LG). | | | | | | | |
| --- | --- | --- | --- | --- | --- | --- | --- |
|  | | | | | | Unique positions | |
| Family | LG | SNP | Length (cM) | Max gap (cM) | Density | SNP | Density |
| J14-3×LC | 1 | 532 | 70.6 | 7.1 | 7.5 | 177 | 2.5 |
| N=73^*^ | 2 | 405 | 96.3 | 5.4 | 4.2 | 151 | 1.6 |
|  | 3 | 342 | 75.8 | 10.3 | 4.5 | 125 | 1.6 |
|  | 4 | 247 | 73.8 | 8.2 | 3.3 | 131 | 1.8 |
|  | 5 | 189 | 107.1 | 11.3 | 1.8 | 110 | 1.0 |
|  | 6 | 386 | 82.3 | 11.2 | 4.7 | 178 | 2.2 |
|  | 7 | 256 | 90.7 | 5.1 | 2.8 | 101 | 1.1 |
| Total | | 2,357 | 596.6 | 11.3 | 4.1 | 973 | 1.7 |
| J14-3×VS | 1 | 406 | 84.6 | 14.0 | 4.8 | 147 | 1.7 |
| N=89 | 2 | 362 | 97.1 | 7.9 | 3.7 | 144 | 1.5 |
|  | 3 | 218 | 79.6 | 14.6 | 2.7 | 81 | 1.0 |
|  | 4 | 329 | 71.9 | 8.5 | 4.6 | 153 | 2.1 |
|  | 5 | 192 | 99.5 | 11.1 | 1.9 | 118 | 1.2 |
|  | 6 | 279 | 71.1 | 4.2 | 3.9 | 143 | 2.0 |
|  | 7 | 221 | 79.1 | 6.5 | 2.8 | 101 | 1.3 |
| Total | | 2,007 | 582.9 | 14.6 | 3.5 | 887 | 1.5 |
| J4-6×RF | 1 | 295 | 70.6 | 7.7 | 4.2 | 120 | 1.7 |
| N=68 | 2 | 321 | 95.6 | 8.4 | 3.4 | 134 | 1.4 |
|  | 3 | 196 | 74.4 | 13.8 | 2.6 | 80 | 1.1 |
|  | 4 | 352 | 85.9 | 8.7 | 4.1 | 149 | 1.7 |
|  | 5 | 200 | 108.9 | 5.3 | 1.8 | 100 | 0.9 |
|  | 6 | 243 | 69.5 | 3.3 | 3.5 | 131 | 1.9 |
|  | 7 | 196 | 75.8 | 6.1 | 2.6 | 98 | 1.3 |
| Total | | 1,803 | 580.7 | 13.8 | 3.2 | 812 | 1.4 |
| OB×J3-6 | 1 | 515 | 87.7 | 17.1 | 5.9 | 183 | 2.1 |
| N=97 | 2 | 242 | 83.4 | 9.4 | 2.9 | 94 | 1.1 |
|  | 3 | 147 | 64.1 | 6.9 | 2.3 | 65 | 1.0 |
|  | 4 | 214 | 71.4 | 9.8 | 3.0 | 108 | 1.5 |
|  | 5 | 378 | 97.9 | 5.5 | 3.9 | 203 | 2.1 |
|  | 6 | 311 | 75.6 | 6.7 | 4.1 | 165 | 2.2 |
|  | 7 | 104 | 36.0 | 3.5 | 2.9 | 52 | 1.4 |
| Total | | 1,911 | 516.1 | 17.1 | 3.6 | 870 | 1.6 |
| OB×RF | 1 | 222 | 70.6 | 8.6 | 3.1 | 69 | 1.0 |
| N=88 | 2 | 401 | 94.6 | 11.9 | 4.2 | 154 | 1.6 |
|  | 3 | 114 | 74.6 | 13.2 | 1.5 | 56 | 0.8 |
|  | 4 | 316 | 85.9 | 4.3 | 3.7 | 203 | 2.4 |
|  | 5 | 443 | 97.9 | 5.5 | 4.5 | 225 | 2.3 |
|  | 6 | 56 | 103.2 | 28.5 | 0.5 | 52 | 0.5 |
|  | 7 | 137 | 89.4 | 30.8 | 1.5 | 74 | 0.8 |
| Total | | 1,689 | 616.2 | 30.8 | 2.7 | 833 | 1.3 |
| Consensus | 1 | 757 | 87.7 | 14.0 | 8.6 | 252 | 2.9 |
| N=415 | 2 | 779 | 97.1 | 3.8 | 8.0 | 308 | 3.2 |
|  | 3 | 518 | 79.6 | 3.5 | 6.5 | 198 | 2.5 |
|  | 4 | 662 | 85.9 | 4.0 | 7.7 | 319 | 3.7 |
|  | 5 | 625 | 108.9 | 4.8 | 5.7 | 318 | 2.9 |
|  | 6 | 619 | 103.2 | 9.4 | 6.0 | 288 | 2.8 |
|  | 7 | 507 | 90.7 | 4.9 | 5.6 | 213 | 2.3 |
| Total | | 4,467 | 653.1 | 14.0 | 6.9 | 1,896 | 2.9 |
| * Number of individuals  LC = ‘Little Chief’, VS=’Vineyard Song’, RF=‘Red Fairy’, OB=’Old Blush’, and SC=’Sweet Chariot’ | | | | | | | |

| **Supplementary Table 3**. Statistical summary of the three individual diploid rose maps and the integrated consensus map (TX2WSE ICM) by linkage group (LG). | | | | | | | |
| --- | --- | --- | --- | --- | --- | --- | --- |
|  | | | | | | Unique positions | |
| Family | LG | SNP | Length (cM) | Max gap (cM) | Density | SNP | Density |
| J14-3×PH | 1 | 949 | 95.7 | 3.2 | 9.9 | 876 | 9.2 |
| N=138^*^ | 2 | 1,179 | 146.1 | 2.6 | 8.1 | 1,076 | 7.4 |
|  | 3 | 1,093 | 94.1 | 4.2 | 11.6 | 976 | 10.4 |
|  | 4 | 1,017 | 93.6 | 1.8 | 10.9 | 919 | 9.8 |
|  | 5 | 1,474 | 130.6 | 2.9 | 11.3 | 1,356 | 10.4 |
|  | 6 | 1,713 | 89.0 | 3.2 | 19.2 | 1,358 | 15.3 |
|  | 7 | 871 | 110.4 | 1.9 | 7.9 | 801 | 7.3 |
| Total | | 8,296 | 759.5 | 4.2 | 10.9 | 7,362 | 9.7 |
| T7-20×SE | 1 | 390 | 75.8 | 1.6 | 5.1 | 359 | 4.7 |
| N=94 | 2 | 677 | 97.6 | 5.5 | 6.9 | 561 | 5.7 |
|  | 3 | 688 | 87.2 | 2.5 | 7.9 | 604 | 6.9 |
|  | 4 | 506 | 78.2 | 2.2 | 6.5 | 458 | 5.9 |
|  | 5 | 816 | 107.6 | 2.2 | 7.6 | 757 | 7.0 |
|  | 6 | 1,125 | 94.5 | 3.0 | 11.9 | 887 | 9.4 |
|  | 7 | 816 | 87.3 | 3.2 | 9.3 | 733 | 8.4 |
| Total | | 5,018 | 628.2 | 5.5 | 8.0 | 4,359 | 6.9 |
| T7-30×SE | 1 | 677 | 97.0 | 1.9 | 7.0 | 619 | 6.4 |
| N=82 | 2 | 1,149 | 146.5 | 5.7 | 7.8 | 999 | 6.8 |
|  | 3 | 730 | 95.5 | 3.1 | 7.6 | 660 | 6.9 |
|  | 4 | 565 | 88.5 | 5.9 | 6.4 | 510 | 5.8 |
|  | 5 | 748 | 104.7 | 2.8 | 7.1 | 694 | 6.6 |
|  | 6 | 1,141 | 103.8 | 2.3 | 11.0 | 953 | 9.2 |
|  | 7 | 954 | 119.9 | 2.8 | 8.0 | 877 | 7.3 |
| Total | | 5,964 | 755.8 | 5.9 | 7.9 | 5,312 | 7.0 |
| Consensus | 1 | 346 | 97.7 | 3.4 | 3.5 | 168 | 1.7 |
| N=314 | 2 | 467 | 146.1 | 4.5 | 3.2 | 197 | 1.3 |
|  | 3 | 345 | 93.9 | 3.4 | 3.7 | 141 | 1.5 |
|  | 4 | 321 | 93.6 | 4.5 | 3.4 | 139 | 1.5 |
|  | 5 | 417 | 123.6 | 5.9 | 3.4 | 194 | 1.6 |
|  | 6 | 389 | 92.8 | 4.2 | 4.2 | 178 | 1.9 |
|  | 7 | 392 | 110.6 | 6.8 | 3.5 | 145 | 1.3 |
| Total | | 2,677 | 758.2 | 6.8 | 3.5 | 1,162 | 1.5 |
| * Number of individuals  PH = ‘Papa Hemeray’, T7-20= TAMU7-20, SE=’Sweet Chariot’, and T7-30= TAMU7-30 | | | | | | | |

| **Supplementary Table 4**. Descriptive statistics of prickle density on the stem and rachis of two multi-parental diploid populations TX2WOB and TX2WSE, in Somerville, Texas, in 2021. | | | | | | |
| --- | --- | --- | --- | --- | --- | --- |
| Trait | Population | N | Mean | Var | Min | Max |
| Stem | TX2WOB | 290 | 9.1 | 31.33 | 0.0 | 24.5 |
|  | TX2WSE | 304 | 9.9 | 48.28 | 0.0 | 30.3 |
| Rachis | TX2WOB | 292 | 1.7 | 2.54 | 0.0 | 10.0 |
|  | TX2WSE | 306 | 3.9 | 7.03 | 0.0 | 17.0 |

| **Supplementary Table 5**. Correlation coefficient (r) between stem and rachis for prickle density phenotyped in Somerville, Texas, on TX2WOB and TX2WSE in 2021. | |
| --- | --- |
| TX2WOB | 0.15** |
| TX2WSE | 0.19** |

| **Supplementary Table 6**. Phenotypic characteristics of TX2WOB progeny regarding prickles (stem/rachis) presence (+) or absence (-) at three loci (*q*SPCK.TX2WOB-LG3.1, *q*SPCK.TX2WOB-LG3.2, and *q*RPCK.TX2WOB-LG6) and associated haplotypes. | | | | | |
| --- | --- | --- | --- | --- | --- |
| **Progeny** | **Stem prickles** | **Rachis prickles** | **Stem haplotypes (LG3.1)** | **Stem haplotypes (LG3.2)** | **Rachis haplotypes (LG6)** |
| SC×M4-4-A0747 | − | − | A5A7 | B5B3 | E4E7 |
| SC×M4-4-A0754 | − | − | A5A7 | B5B3 | E4E7 |
| J14-3×SC-A2025 | − | − | A3A5 | NA | E6E3 |
| SC×M4-4-A0730 | − | NA | A5A4 | B5B3 | NA |
| SC×M4-4-A0750 | − | + | A5A4 | B5B3 | E4E7 |
| SC×M4-4-A0762 | − | NA | A5A7 | B5B3 | NA |
| SC×M4-4-D3105 | − | − | A5A7 | B5B3 | E3E7 |
| J14-3×LC-1449 | + | − | A3A6 | B3B1 | E6E1 |
| J14-3×LC-1450 | + | − | A3A2 | B3B1 | E5E3 |
| J14-3×LC-1508 | + | − | A1A2 | NA | E5E3 |
| J14-3×LC-1522 | + | − | A3A2 | B3B2 | E5E3 |
| J14-3×LC-1526 | + | − | A1A6 | B4B1 | E5E3 |
| J14-3×LC-1535 | + | − | A3A2 | B3B2 | E5E1 |
| J14-3×LC-1541 | + | − | A1A6 | B3B1 | E6E3 |
| J14-3×LC-1617 | + | − | A3A6 | B3B1 | E5E3 |
| J14-3×SC-A2005 | + | − | A1A6 | B4B5 | E5E4 |
| J14-3×SC-A2009 | + | − | A3A5 | B3B5 | E5E3 |
| J14-3×SC-A2010 | + | − | A3A5 | B3B5 | E5E4 |
| J14-3×SC-A2021 | + | − | A1A5 | B4B5 | E5E4 |
| J14-3×SC-A2029 | + | − | A1A5 | B4B5 | E6E4 |
| J14-3×SC-A2033 | + | − | A1A5 | B3B5 | E5E3 |
| J14-3×SC-A2054 | + | − | A1A5 | B4B5 | E5E4 |
| J14-3×SC-D3544 | + | − | A3A6 | B3B1 | E5E4 |
| J14-3×SC-D3549 | + | − | A3A6 | B3B1 | E5E4 |
| J14-3×SC-D3601 | + | − | A1A6 | NA | E5E3 |
| J14-3×SC-D3605 | + | − | A1A5 | B4B5 | E5E4 |
| J14-3×VS-1842 | NA | − | NA | NA | E5E1 |
| OB×M4-4-A1326 | − | NA | NA | B3B3 | NA |
| SC×J14-3-D3518 | + | − | A5A1 | B5B4 | E3E5 |
| SC×J14-3-D3531 | + | − | A5A1 | B5B4 | E3E5 |
| SC×J14-3-D3538 | + | − | A6A1 | B1B4 | E3E5 |
| SC×J14-3-D3539 | + | − | A6A1 | B1B4 | E4E5 |
| SC×J14-3-D3541 | + | − | A6A1 | NA | E3E6 |
| SC×M4-4-A0719 | + | − | A5A4 | B5B3 | E4E7 |
| SC×M4-4-A0739 | + | − | A5A7 | B5B3 | E4E7 |
| SC×M4-4-A0752 | + | − | A6A7 | B1B3 | E4E7 |
| SC×M4-4-D3106 | + | − | A6A4 | B1B3 | E4E7 |
| SC×M4-4-D3109 | + | − | A5A7 | B5B3 | E4E7 |
| J14-3×SC-A2019 | + | − | NA | B4B5 | E6E4 |
| J14-3×SC-A2050 | + | − | NA | B3B1 | E5E4 |
| J14-3×SC-A2055 | + | − | NA | B4B5 | E5E3 |
| J14-3×SC-D3551 | + | − | NA | B3B1 | E6E4 |
| J14-3×SC-D3604 | + | − | NA | B4B5 | E5E4 |
| OB×M4-4-A1320 | − | NA | NA | B3B3 | NA |
| OB×M4-4-A1335 | − | NA | NA | B3B3 | NA |
| SC×J14-3-D3526 | + | − | NA | B5B4 | E3E5 |
| J14-3×RF-D1825 | NA | − | NA | NA | E6E2 |
| J14-3×RF-D1831 | NA | − | NA | NA | E5E2 |
| J14-3×RF-D1840 | NA | − | NA | NA | E5E2 |
| J14-3×RF-D1841 | NA | − | NA | NA | E5E2 |
| J14-3×RF-D1842 | NA | − | NA | NA | E5E2 |
| J14-3×RF-D1845 | NA | − | NA | NA | E5E2 |
| J14-3×RF-D1847 | NA | − | NA | NA | E6E2 |
| J14-3×RF-D1849 | NA | − | NA | NA | E5E2 |
| J14-3×RF-D1850 | NA | − | NA | NA | E5E2 |
| J14-3×RF-D1913 | NA | − | NA | NA | E6E2 |
| J14-3×RF-D1915 | NA | − | NA | NA | E5E2 |
| J14-3×RF-D1919 | NA | − | NA | NA | E5E2 |
| J14-3×RF-D1923 | NA | − | NA | NA | E5E2 |
| J14-3×RF-D1926 | NA | − | NA | NA | E5E2 |
| J14-3×RF-D1929 | NA | − | NA | NA | E5E2 |
| J14-3×RF-D1937 | NA | − | NA | NA | E6E2 |
| J14-3×VS-1839 | NA | − | NA | NA | E5E1 |
| J14-3×VS-1852 | NA | − | NA | NA | E6E1 |
| J14-3×VS-1858 | NA | − | NA | NA | E6E1 |
| J14-3×VS-1869 | NA | − | NA | NA | E6E1 |
| J14-3×VS-1874 | NA | − | NA | NA | E6E1 |
| J14-3×VS-1877 | NA | − | NA | NA | E5E1 |
| J14-3×VS-1921 | NA | − | NA | NA | E5E1 |
| J14-3×VS-1928 | NA | − | NA | NA | E5E1 |
| J14-3×VS-1964 | NA | − | NA | NA | E6E1 |
| NA = Data not available | | | | | |

| **Supplementary Table 7**. Phenotypic characteristics of TX2WSE progeny regarding prickles (stem/rachis) presence (+) or absence (-) at three loci (*q*SPCK.TX2WSE-LG3.1, *q*SPCK.TX2WSE-LG3.2, and *q*RPCK.TX2WSE-LG3) and associated haplotypes. | | | | | |
| --- | --- | --- | --- | --- | --- |
| **Progeny** | **Stem prickles** | **Rachis prickles** | **Stem haplotypes (LG3.1)** | **Stem haplotypes (LG3.2)** | **Rachis haplotypes (LG3)** |
| M4.4×SE.681.21 | **−** | **−** | C5C3 | D5D7 | F5F3 |
| T7.20×SE.685.103 | **−** | **−** | C5C1 | D5D7 | F5F1 |
| T7.30×SE.686.4 | **−** | **−** | C6C3 | D8D7 | F6F3 |
| J14.3×PH.547.24 | **−** | **−** | C2C5 | NA | F2F5 |
| J14.3×PH.547.25 | **−** | **−** | C1C5 | NA | F1F5 |
| J14.3×PH.547.53 | **−** | **−** | C2C5 | NA | F2F5 |
| J14.3×PH.547.71 | **−** | **−** | C2C5 | NA | F2F5 |
| J14.3×PH.649.4 | **−** | **−** | C1C5 | NA | F1F5 |
| J14.3×PH.649.29 | **−** | **−** | C2C5 | NA | F2F5 |
| M4.4×SE.681.1 | **−** | **+** | C5C1 | D5D7 | F5F1 |
| M4.4×SE.681.2 | **−** | **+** | C5C1 | D5D7 | F5F1 |
| M4.4×SE.681.4 | **−** | **+** | C5C3 | D5D7 | F5F3 |
| M4.4×SE.681.8 | **−** | **+** | C5C1 | D8D7 | F5F1 |
| M4.4×SE.681.9 | **−** | **+** | C5C1 | D5D7 | F5F1 |
| M4.4×SE.681.12 | **−** | **+** | C5C3 | D8D7 | F5F3 |
| M4.4×SE.681.17 | **−** | **+** | C5C3 | D5D7 | F5F3 |
| M4.4×SE.681.22 | **−** | **+** | C5C3 | D8D7 | F5F3 |
| M4.4×SE.681.29 | **−** | **+** | C5C3 | D5D7 | F5F3 |
| M4.4×SE.681.32 | **−** | **+** | C5C3 | D8D7 | F5F3 |
| M4.4×SE.681.33 | **−** | **+** | C5C3 | D8D7 | F5F3 |
| T7.20×SE.685.2 | **−** | **+** | C5C3 | D5D7 | F5F3 |
| T7.20×SE.685.9 | **−** | **+** | C6C3 | D5D7 | F6F3 |
| T7.20×SE.685.10 | **−** | **+** | C6C1 | D5D7 | F6F1 |
| T7.20×SE.685.12 | **−** | **+** | C6C3 | D5D7 | F6F3 |
| T7.20×SE.685.17 | **−** | **+** | C5C3 | D5D7 | F5F3 |
| T7.20×SE.685.22 | **−** | **+** | C6C3 | D5D7 | F6F3 |
| T7.20×SE.685.30 | **−** | **+** | C5C3 | D5D7 | F5F3 |
| T7.20×SE.685.35 | **−** | **+** | C6C3 | D5D7 | F6F3 |
| T7.20×SE.685.36 | **−** | **+** | C6C1 | D5D7 | F6F1 |
| T7.20×SE.685.43 | **−** | **+** | C5C3 | D5D7 | F5F3 |
| T7.20×SE.685.53 | **−** | **+** | C6C1 | D5D7 | F6F1 |
| T7.20×SE.685.80 | **−** | **+** | C6C3 | D5D7 | F6F3 |
| T7.20×SE.685.99 | **−** | **+** | C5C1 | D5D7 | F5F1 |
| T7.30×SE.686.3 | **−** | **+** | C6C3 | D4D7 | F6F3 |
| T7.30×SE.686.5 | **−** | **+** | C5C3 | D8D7 | F5F3 |
| T7.30×SE.686.6 | **−** | **+** | C6C3 | D4D7 | F6F3 |
| T7.30×SE.686.9 | **−** | **+** | C5C3 | D8D7 | F5F3 |
| T7.30×SE.686.10 | **−** | **+** | C5C3 | D8D7 | F5F3 |
| T7.30×SE.686.13 | **−** | **+** | C6C1 | D4D7 | F6F1 |
| T7.30×SE.686.18 | **−** | **+** | C5C3 | D8D7 | F5F3 |
| T7.30×SE.686.30 | **−** | **+** | C5C1 | D8D7 | F5F1 |
| T7.30×SE.686.40 | **−** | **+** | C6C1 | D8D7 | F6F1 |
| T7.30×SE.686.60 | **−** | **+** | C5C3 | D8D7 | F5F3 |
| T7.30×SE.686.61 | **−** | **+** | C6C1 | D8D7 | F6F1 |
| T7.30×SE.686.64 | **−** | **+** | C6C1 | D8D7 | F6F1 |
| T7.30×SE.686.71 | **−** | **−** | C6C3 | NA | F6F3 |
| T7.30×SE.686.73 | **−** | **+** | C5C3 | D8D7 | F5F3 |
| T7.30×SE.686.86 | **−** | **+** | C5C1 | D8D7 | F5F1 |
| T7.30×SE.686.89 | **−** | **+** | C6C1 | D8D7 | F6F1 |
| J14.3×PH.547.5 | **+** | **−** | C1C5 | NA | F1F5 |
| J14.3×PH.547.14 | **+** | **−** | C1C5 | NA | F1F5 |
| J14.3×PH.547.19 | **+** | **−** | C1C5 | NA | F1F5 |
| J14.3×PH.547.35 | **+** | **−** | C1C5 | NA | F1F5 |
| J14.3×PH.547.36 | **+** | **−** | C2C5 | NA | F2F5 |
| J14.3×PH.547.39 | **+** | **−** | C2C5 | NA | F2F5 |
| J14.3×PH.547.49 | **+** | **−** | C2C5 | NA | F2F5 |
| J14.3×PH.547.51 | **+** | **−** | C1C5 | NA | F1F5 |
| J14.3×PH.547.59 | **+** | **−** | C1C5 | NA | F1F5 |
| J14.3×PH.547.61 | **+** | **−** | C1C5 | NA | F1F5 |
| J14.3×PH.547.67 | **+** | **−** | C1C5 | NA | F1F5 |
| J14.3×PH.547.68 | **+** | **−** | C1C5 | NA | F1F5 |
| J14.3×PH.547.70 | **+** | **−** | C1C5 | NA | F1F5 |
| J14.3×PH.547.73 | **+** | **−** | C1C5 | NA | F1F5 |
| J14.3×PH.547.75 | **+** | **−** | C2C5 | NA | F2F5 |
| J14.3×PH.649.1 | **+** | **−** | C2C5 | NA | F2F5 |
| J14.3×PH.649.6 | **+** | **−** | C1C5 | NA | F1F5 |
| J14.3×PH.649.8 | **+** | **−** | C2C5 | NA | F2F5 |
| J14.3×PH.649.15 | **+** | **−** | C1C5 | NA | F1F5 |
| J14.3×PH.649.17 | **+** | **−** | C2C5 | NA | F2F5 |
| J14.3×PH.649.18 | **+** | **−** | C2C5 | NA | F2F5 |
| J14.3×PH.649.22 | **+** | **−** | C1C5 | NA | F1F5 |
| J14.3×PH.649.25 | **+** | **−** | C1C5 | NA | F1F5 |
| J14.3×PH.649.26 | **+** | **−** | C1C5 | NA | F1F5 |
| J14.3×PH.649.27 | **+** | **−** | C2C5 | NA | F2F5 |
| J14.3×PH.649.31 | **+** | **−** | C1C5 | NA | F1F5 |
| J14.3×PH.649.32 | **+** | **−** | C1C5 | NA | F1F5 |
| J14.3×PH.649.37 | **+** | **−** | C2C5 | NA | F2F5 |
| J14.3×PH.649.42 | **+** | **−** | C1C5 | NA | F1F5 |
| J14.3×PH.649.51 | NA | **−** | NA | NA | F2F5 |
| J14.3×PH.649.53 | **+** | **−** | C1C5 | NA | F1F5 |
| J14.3×PH.649.54 | **+** | **−** | C1C5 | NA | F1F5 |
| J14.3×PH.649.55 | **+** | **−** | C2C5 | NA | F2F5 |
| J14.3×PH.649.62 | **+** | **−** | C1C5 | NA | F1F5 |
| J14.3×PH.649.68 | **+** | **−** | C2C5 | NA | F2F5 |
| J14.3×PH.649.74 | **+** | **−** | C1C5 | NA | F1F5 |
| J14.3×PH.649.77 | NA | **−** | NA | NA | F1F5 |
| SET.ARE×OL.407.15 | **+** | **−** | C4C6 | NA | F4F6 |
| T7.20×SE.685.3 | **+** | **−** | C5C3 | NA | F5F3 |
| T7.20×SE.685.46 | **+** | **−** | C6C3 | D1D7 | F6F3 |
| T7.20×SE.685.69 | **+** | **−** | C5C1 | D5D7 | F5F1 |
| T7.30×SE.686.14 | **+** | **−** | C6C3 | D4D2 | F6F3 |
| T7.30×SE.686.16 | **+** | **−** | C6C3 | D4D7 | F6F3 |
| T7.30×SE.686.22 | **−** | **+** | C6C1 | NA | F6F1 |
| T7.30×SE.686.31 | **−** | **+** | C6C3 | NA | F6F3 |
| T7.30×SE.686.53 | **−** | **+** | C5C3 | NA | F5F3 |
| PH×SEB.ARE.660.3 | **−** | NA | NA | D6D3 | NA |
| T7.20×SE.685.76 | **−** | NA | NA | D5D7 | NA |
| NA = Data not available | | | | | |
